# Cdc14 phosphatase directs centrosome re-duplication at the meiosis I to meiosis II transition in budding yeast

**DOI:** 10.12688/wellcomeopenres.10507.2

**Published:** 2017-02-21

**Authors:** Colette Fox, Juan Zou, Juri Rappsilber, Adele L. Marston

**Affiliations:** 1 The Wellcome Trust Centre for Cell Biology, Institute of Cell Biology, School of Biological Sciences, Edinburgh, UK; 2 Chair of Bioanalytics, Institute of Biotechnology, Technische Universität Berlin, Berlin, Germany

**Keywords:** Meiosis, Cdc14, SPB, centrosome

## Abstract

*Background*: Gametes are generated through a specialized cell division called meiosis, in which ploidy is reduced by half because two consecutive rounds of chromosome segregation, meiosis I and meiosis II, occur without intervening DNA replication. This contrasts with the mitotic cell cycle where DNA replication and chromosome segregation alternate to maintain the same ploidy. At the end of mitosis, cyclin-dependent kinases (CDKs) are inactivated. This low CDK state in late mitosis/G1 allows for critical preparatory events for DNA replication and centrosome/spindle pole body (SPB) duplication. However, their execution is inhibited until S phase, where further preparatory events are also prevented. This “licensing” ensures that both the chromosomes and the centrosomes/SPBs replicate exactly once per cell cycle, thereby maintaining constant ploidy. Crucially, between meiosis I and meiosis II, centrosomes/SPBs must be re-licensed, but DNA re-replication must be avoided. In budding yeast, the Cdc14 protein phosphatase triggers CDK down regulation to promote exit from mitosis. Cdc14 also regulates the meiosis I to meiosis II transition, though its mode of action has remained unclear.

*Methods*: Fluorescence and electron microscopy was combined with proteomics to probe SPB duplication in cells with inactive or hyperactive Cdc14.

*Results*: We demonstrate that Cdc14 ensures two successive nuclear divisions by re-licensing SPBs at the meiosis I to meiosis II transition. We show that Cdc14 is asymmetrically enriched on a single SPB during anaphase I and provide evidence that this enrichment promotes SPB re-duplication. Cells with impaired Cdc14 activity fail to promote extension of the SPB half-bridge, the initial step in morphogenesis of a new SPB. Conversely, cells with hyper-active Cdc14 duplicate SPBs, but fail to induce their separation.

*Conclusion*: Our findings implicate reversal of key CDK-dependent phosphorylations in the differential licensing of cyclical events at the meiosis I to meiosis II transition.

## Introduction

Meiosis is a specialized cell division, which generates gametes. In the canonical mitotic cell cycle, ploidy is maintained by alternating S and M phases. In contrast, during meiosis, chromosome duplication in S phase is followed by two consecutive chromosome segregation phases, meiosis I and meiosis II, to generate gametes with half the ploidy of the parental cell. Therefore, in addition to distinct modifications to the chromosome segregation machinery, meiosis requires a re-wiring of cell cycle controls (reviewed in Duro & Marston, 2015). Progression through the cell cycle is driven by cyclin-dependent kinases (CDKs), in association with distinct cyclin subunits. CDK activity is low in G1, but upon cell cycle entry, activation of S-phase and mitotic CDKs in turn promote DNA replication followed by spindle assembly and chromosome segregation. Following completion of chromosome segregation, CDKs are inactivated, triggering spindle disassembly and the return to G1 (mitotic exit) ( Stegmeier & Amon, 2004). This state of low CDK activity in G1 allows for the re-licensing of DNA replication origins and centrosomes/spindle pole bodies (SPBs), events that must be restricted to once per cell cycle. In budding yeast, the Cdc14 phosphatase triggers CDK inactivation through multiple mechanisms to promote exit from mitosis and return to G1 ( Jaspersen *et al*., 1998; Visintin *et al*., 1998; Zachariae *et al*., 1998). Cdc14 is regulated by its localization: for the majority of the cell cycle it is sequestered in the nucleolus through association with its inhibitor, Cfi1/Net1 ( Shou *et al*., 1999; Visintin *et al*., 1999). Upon chromosome segregation at anaphase onset, the Cdc *F*ourteen *E*arly *A*naphase *R*elease (FEAR) network promotes Cdc14 release from the nucleolus into the nucleus; later in anaphase the *M*itotic *E*xit *N*etwork (MEN) maintains Cdc14 release throughout the cytoplasm ( Pereira *et al*., 2002a; Stegmeier & Amon, 2004; Yoshida *et al*., 2002). While FEAR-dependent Cdc14 release promotes successful completion of chromosome segregation, only MEN-dependent Cdc14 release is sufficient to trigger exit from mitosis, leading to spindle disassembly and entry into G1 ( Yellman & Roeder, 2015).

The state of low CDK activity in G1 is permissive for the assembly of pre-replicative complexes, the later firing of which requires S phase CDKs (reviewed in ( Blow & Dutta, 2005; Drury & Diffley, 2009)). This separation of pre-RC assembly and firing into differential CDK activity states ensures that DNA replication is strictly restricted to once per cell cycle. Similarly, centrosome/SPB duplication must occur exactly once in each cell cycle. Yeast SPBs are microtubule-organising centres, composed of at least 19 proteins, forming three layers that are assembled into a cylindrical organelle embedded in the nuclear envelope (reviewed in Jaspersen & Winey, 2004). SPB duplication is initiated in G1 by extension of the half-bridge ( Byers & Goetsch, 1974; Vallen *et al*., 1994), which protrudes from the central SPB layer and consists of Kar1, Mps3, Sfi1 and Cdc31. The full bridge structure serves as the site for new SPB assembly ( Jaspersen *et al*., 2002; Kilmartin, 2003; Spang *et al*., 1993; Spang *et al*., 1995), so that at the close of G1, two side-by-side SPBs are physically connected by a full-bridge ( Byers & Goetsch, 1974). S phase CDK activity severs the bridge structure triggering SPB separation. Recent work has identified the bridge component Sfi1 as the target of S phase CDKs ( Avena *et al*., 2014; Elserafy *et al*., 2014). S phase CDKs also activate the Cin8 and Kip1 motors that drive separation of the SPBs to enable bipolar spindle formation ( Crasta *et al*., 2006; Crasta *et al*., 2008; Haase *et al*., 2001). In mitosis, M phase CDKs prevent SPB re-duplication through phosphorylation of Sfi1, thereby preventing the initiation of half-bridge elongation ( Avena *et al*., 2014; Bouhlel *et al*., 2015; Elserafy *et al*., 2014; Haase *et al*., 2001). For SPBs to re-duplicate in the following G1-phase, Sfi1 must be dephosphorylated and Clb-CDKs inhibited. Recent findings have implicated Cdc14 as the phosphatase responsible for this dephosphorylation event in budding yeast ( Avena *et al*., 2014; Elserafy *et al*., 2014).

The meiosis I to meiosis II transition requires specialized cell cycle controls. Uniquely, chromosome segregation at meiosis I exit is followed not by a DNA replication phase, but by a second chromosome segregation phase, meiosis II. Importantly, centrosome/SPBs must be re-licensed at meiosis I exit to permit this additional segregation event, yet DNA replication origins must not be re-set to avoid over-duplication of chromosomes. How this is controlled is not clear. One potential contributing factor is the retention of partial CDK activity between meiosis I and meiosis II. Indeed, there is evidence to suggest that CDKs are only partially downgraded between meiosis I and II in *Xenopus* oocytes and fission yeast ( Iwabuchi *et al*., 2000; Izawa *et al*., 2005). However, how these global alterations in CDK activity impinge on the differential re-licensing of DNA replication origins and SPBs has not been investigated.

In budding yeast, the Cdc14 phosphatase plays a prominent role in the meiosis I to meiosis II transition ( Buonomo *et al*., 2003; Marston *et al*., 2003). Following meiosis I chromosome segregation, *cdc14-1* mutants disassemble the spindle, only to reassemble a single spindle that directs segregation of some chromosomes in a meiosis II-like manner ( Bizzari & Marston, 2011). The result is binucleate, rather than tetranucleate, cells with a mixed complement of chromosomes ( Sharon & Simchen, 1990). Furthermore, ectopic activation of Cdc14 is also detrimental to meiosis. Depletion of the regulatory subunit of protein phosphatase 2A, Cdc55, results in premature release of Cdc14 from the nucleolus in meiosis and a block to spindle assembly, so that nuclear division largely fails ( Bizzari & Marston, 2011; Kerr *et al*., 2011; Nolt *et al*., 2011). Inactivation of Cdc14 in Cdc55-depleted cells enables spindle assembly and the production of binucleate cells, indicating that over-active Cdc14 is responsible for the block to spindle assembly ( Bizzari & Marston, 2011; Kerr *et al*., 2011). Therefore, proper regulation of Cdc14 is critical to control spindle morphogenesis during meiosis, but not the dissolution of linkages between chromosomes. While the FEAR network plays a vital role in the release of Cdc14 in anaphase I, MEN is dispensable ( Buonomo *et al*., 2000; Kamieniecki *et al*., 2005; Marston *et al*., 2003; Pablo-Hernando *et al*., 2007) and does not appear to be active until anaphase II ( Attner & Amon, 2012). Since FEAR-dependent Cdc14 release appears insufficient to trigger CDK inactivation ( Stegmeier *et al*., 2002; Yellman & Roeder, 2015), it is likely that the critical role of Cdc14 at the meiosis I to meiosis II transition is to reverse the phosphorylation of key substrates.

Here we investigate the role of Cdc14 in executing the meiosis I to meiosis II transition. Our findings suggest that a critical role of Cdc14 at meiosis I exit is to re-license SPB duplication. This re-licensing ensures assembly of a pair of spindles for a second round of nuclear division at meiosis II. Conversely, premature Cdc14 activation prevents SPB separation. We provide evidence that Cdc14 associates with the SPB in meiosis and that this localization is important for permitting the duplication cycle. Our data suggest that the critical function of Cdc14 at the meiosis I to meiosis II transition is to reverse key phosphorylations to enable SPB re-duplication.

## Materials and methods

### Yeast strains and plasmids

Yeast strains used in this study were generated using standard genetic methods and are given in Table 1. *pCLB2-3HA-CDC55* ( Clift *et al*., 2009), *CLB1-9MYC* ( Buonomo *et al*., 2003), *pCLB2-CDC20* ( Lee & Amon, 2003), *GAL-NDT80* and *pGPD1-GAL4(848). ER* ( Benjamin *et al*., 2003), *cdc14-1, slk19Δ* and *spo12Δ* ( Marston *et al*., 2003) *CDC14-GFP, PDS1-tdTomato* and *GFP-TUB1* ( Matos *et al*., 2008) were as described. *SPC42-*tdTomato, *CDC14-SZZ(TAP), SPC42-3FLAG, SPC42-GFP, BFA1-tdTomato, bub2Δ, bfa1Δ, kin4Δ* and *bmh1Δ* were made using a one-step PCR method ( Longtine *et al*., 1998). The S *PC42-CFP* strain was obtained by integrating the pHX144 plasmid at the *SPC42* locus ( He *et al*., 2000).

### Growth conditions

To induce meiosis, diploid strains were removed from -80°C storage onto YPG (2% Bacto-peptone, 1% Bacto-yeast extract, 2.5% glycerol) plates and grown overnight (~16 h). The following day, cells were patched to 4%YPDA (2% Bacto-peptone, 1% Bacto-yeast extract, 4% glucose, 0.3 mM adenine) plates. On the third consecutive day, YPDA (2% Bacto-peptone, 1% Bacto-yeast extract, 2% glucose, 0.3 mM adenine) liquid cultures were inoculated with yeast strains and grown overnight. Cells were then diluted to OD _600_ =0.2 in BYTA (2% Bacto-peptone, 1% Bacto-yeast extract, 1% Potassium acetate, 50 mM Potassium Phthalate) liquid culture and grown to OD _600_ = 6-10. On the fifth day, cells were washed once in sterile water and resuspended in SPO liquid media (0.3 % Potassium acetate, pH 7.0) at OD _600_ = 1.8-3. Meiosis was performed at 30°C. For experiments with temperature sensitive *cdc14-1* mutants, all steps were performed at room temperature prior to resuspension in SPO medium, upon which cultures were shifted to 30°C and incubated at this temperature for the remainder of the experiment.

**Table 1.** Yeast strains used in the present study.

Strain number	Genotype
AM1835	*MATa/MATα* Wild type
AM9319	*MATa/MATα* *CDC14-SZZ(TAP)::KanMX6/CDC14-SZZ(TAP)::KanMX6 cdc55::KanMX6::PCLB2-* *3HA-CDC55/cdc55::KanMX6::PCLB2-3HA-CDC55*
AM9434	*MATa/MATα* *CDC14-SZZ(TAP)::KanMX6/CDC14-SZZ(TAP)::KanMX6*
AM9459	*MATa/MATα* *cdc55::KanMX6::PCLB2-3HA-CDC55/cdc55::KanMX6::PCLB2-3HA-CDC55*
AM11443	*MATa/MATα* *SPC42-3FLAG-KANMX6/SPC42-3FLAG-KANMX6 cdc14::KanMX6/cdc14::KanMX6* *trp1::cdc14-1::TRP1::LEU2/trp1::cdc14-1::TRP1::LEU2*
AM11444	*MATa/MATα* *SPC42-3FLAG-KANMX6/SPC42-3FLAG-KANMX6*
AM11517	*MATa/MATα* *CDC14-GFP-LEU2/CDC14-GFP-LEU2* *SPC42-tdTomato::NAT/SPC42-tdTomato::NAT* *GAL-NDT80::TRP1/GAL-NDT80::TRP1* *ura3::pGPD1-GAL4(848).ER::URA3/ura3::pGPD1-GAL4(848).ER::URA3*
AM13123	*MATa/MATα* *CDC14-GFP-LEU2/CDC14-GFP-LEU2* *SPC42-tdTomato::NAT/SPC42-tdTomato::NAT* *cdc55::KanMX6::PCLB2-3HA-CDC55/cdc55::KanMX6::PCLB2-3HA-CDC55* *GAL-NDT80::TRP1/GAL-NDT80::TRP1* *ura3::pGPD1-GAL4(848).ER::URA3/ura3::pGPD1-GAL4(848).ER::URA3*
AM13989	*MATa/MATα* *SPC42-tdTomato::NAT/SPC42-tdTomato::NAT* *GAL-NDT80::TRP1/GAL-NDT80::TRP1* *ura3::pGPD1-GAL4(848).ER::URA3/ura3::pGPD1-GAL4(848).ER::URA3*
AM15543	*MATa/MATα* *CDC14-GFP-LEU2/CDC14-GFP-LEU2* *SPC42-tdTomato::NAT/SPC42-tdTomato::NAT* *bub2Δ::NAT/bub2Δ::NAT* *GAL-NDT80::TRP1/GAL-NDT80::TRP1* *ura3::pGPD1-GAL4(848).ER::URA3/ura3::pGPD1-GAL4(848).ER::URA3*
AM15984	*MATa/MATα* *SPC42-tdTomato::NAT/SPC42-tdTomato::NAT* *cdc55::KanMX6::PCLB2-3HA-CDC55/cdc55::KanMX6::PCLB2-3HA-CDC55* *GAL-NDT80::TRP1/ GAL-NDT80::TRP1* *ura3::pGPD1-GAL4(848).ER::URA3/ura3::pGPD1-GAL4(848).ER::URA3*
AM15985	*MATa/MATα* *his3::HIS3p-GFP-TUB1-HIS3/his3::HIS3p-GFP-TUB1-HIS3* *PDS1-tdTomato-KITRP1/PDS1-tdTomato-KITRP1* *spo12Δ::LEU2/spo12Δ::LEU2* *GAL-NDT80::TRP1/GAL-NDT80::TRP1* *ura3::pGPD1-GAL4(848).ER::URA3/ura3::pGPD1-GAL4(848).ER::URA3*
AM16019	*MATa/MATα* *SPC42-tdTomato::NAT/SPC42-tdTomato::NAT* *bfa1Δ::NAT/bfa1Δ::NAT* *GAL-NDT80::TRP1/GAL-NDT80::TRP1* *ura3::pGPD1-GAL4(848).ER::URA3/ura3::pGPD1-GAL4(848).ER::URA3*
AM16020	*MATa/MATα* *SPC42-tdTomato::NAT/SPC42-tdTomato::NAT* *slk19Δ::KANMX6/slk19Δ::KANMX6* *GAL-NDT80::TRP1/GAL-NDT80::TRP1* *ura3::pGPD1-GAL4(848).ER::URA3/ura3::pGPD1-GAL4(848).ER::URA3*
AM16064	*MATa/MATα* *SPC42-tdTomato::NAT/SPC42-tdTomato::NAT* *spo12Δ::LEU2/spo12Δ::LEU2* *GAL-NDT80::TRP1/GAL-NDT80::TRP1* *ura3::pGPD1-GAL4(848).ER::URA3/ura3::pGPD1-GAL4(848).ER::URA3*
AM16065	*MATa/MATα* *his3::HIS3p-GFP-TUB1-HIS3/his3::HIS3p-GFP-TUB1-HIS3* *PDS1-tdTomato-KITRP1/PDS1-tdTomato-KITRP1* *GAL-NDT80::TRP1/GAL-NDT80::TRP1* *ura3::pGPD1-GAL4(848).ER::URA3/ura3::pGPD1-GAL4(848).ER::URA3*
AM16066	*MATa/MATα* *his3::HIS3p-GFP-TUB1-HIS3/his3::HIS3p-GFP-TUB1-HIS3* *PDS1-tdTomato-KITRP1/PDS1-tdTomato-KITRP1* *cdc14::KanMX6/cdc14::KanMX6* *leu2::cdc14-1::LEU2::TRP1/leu2::cdc14-1::LEU2::TRP1* *GAL-NDT80::TRP1/GAL-NDT80::TRP1* *ura3::pGPD1-GAL4(848).ER::URA3/ura3::pGPD1-GAL4(848).ER::URA3*
AM16077	*MATa/MATα* *cdc14::KanMX6/cdc14::KanMX6* *leu2::cdc14-1::LEU2::TRP1/leu2::cdc14-1::LEU2::TRP1* *GAL-NDT80::TRP1/GAL-NDT80::TRP1* *ura3::pGPD1-GAL4(848).ER::URA3/ura3::pGPD1-GAL4(848).ER::URA3*
AM16079	*MATa/MATα* *CDC14-GFP-LEU2/CDC14-GFP-LEU2* *SPC42-tdTomato::NAT/SPC42-tdTomato::NAT* *bfa1Δ::NAT/bfa1Δ::NAT* *GAL-NDT80::TRP1/GAL-NDT80::TRP1* *ura3::pGPD1-GAL4(848).ER::URA3/ura3::pGPD1-GAL4(848).ER::URA3*
AM16080	*MATa/MATα* *SPC42-tdTomato::NAT/SPC42-tdTomato::NAT* *bub2Δ::NAT/bub2Δ::NAT* *GAL-NDT80::TRP1/GAL-NDT80::TRP1* *ura3::pGPD1-GAL4(848).ER::URA3/ura3::pGPD1-GAL4(848).ER::URA3*
AM16110	*MATa/MATα* *his3::HIS3p-GFP-TUB1-HIS3/his3::HIS3p-GFP-TUB1-HIS3* *PDS1-tdTomato-KITRP1/PDS1-tdTomato-KITRP1 slk19Δ::TRP/slk19Δ::TRP* *GAL-NDT80::TRP1/GAL-NDT80::TRP1* *ura3::pGPD1-GAL4(848).ER::URA3/ura3::pGPD1-GAL4(848).ER::URA3*
AM16163	*MATa/MATα* *SPC42-tdTomato::NAT/SPC42-tdTomato::NAT* *cdc14::KanMX6/cdc14::KanMX6* *trp1::cdc14-1::TRP1::LEU2/trp1::cdc14-1::TRP1::LEU2* *GAL-NDT80::TRP1/GAL-NDT80::TRP1* *ura3::pGPD1-GAL4(848).ER::URA3/ura3::pGPD1-GAL4(848).ER::URA3*
AM16198	*MATa/MATα* *cdc55::KanMX6::PCLB2-3HA-CDC55/cdc55::KanMX6::PCLB2-3HA-CDC55* *GAL-NDT80::TRP1/GAL-NDT80::TRP1* *ura3::pGPD1-GAL4(848).ER::URA3/ura3::pGPD1-GAL4(848).ER::URA3*
AM17134	*MATa/MATα* *CDC14-GFP-LEU2/CDC14-GFP-LEU2* *SPC42-tdTomato::NAT/SPC42-tdTomato::NAT* *kin4Δ::NAT/kin4Δ::NAT* *GAL-NDT80::TRP1/GAL-NDT80::TRP1* *ura3::pGPD1-GAL4(848).ER::URA3/ura3::pGPD1-GAL4(848).ER::URA3*
AM17341	*MATa/MATα* *CDC14-GFP-LEU2/CDC14-GFP-LEU2* *SPC42-tdTomato::NAT/SPC42-tdTomato::NAT* *bmh1Δ::NAT/bmh1Δ::NAT* *GAL-NDT80::TRP1/GAL-NDT80::TRP1* *ura3::pGPD1-GAL4(848).ER::URA3/ura3::pGPD1-GAL4(848).ER::URA3*
AM17740	*MATa/MATα* *Bfa1-tdTomato::NAT/Bfa1-tdTomato::NAT* *SPC42-CFP::TRP1/SPC42-CFP::TRP1* *CDC14-GFP-LEU2/CDC14-GFP-LEU2* *ura3::pGPD1-GAL4(848).ER::URA3/ura3::pGPD1-GAL4(848).ER::URA3*
AM17904	*MATa/MATα* *SPC42-tdTomato::NAT/SPC42-tdTomato::NAT* *cdc14::KanMX6/cdc14::KanMX6* *trp1::cdc14-1::TRP1::LEU2/trp1::cdc14-1::TRP1::LEU2* *cdc55::KanMX6::PCLB2-3HA-CDC55/cdc55::KanMX6::PCLB2-3HA-CDC55* *GAL-NDT80::TRP1/GAL-NDT80::TRP1* *ura3::pGPD1-GAL4(848).ER::URA3/ura3::pGPD1-GAL4(848).ER::URA3*

### Fluorescence microscopy

To visualize chromosomes and SPBs labelled with fluorescent proteins in fixed cells, 100 μl of meiotic culture was added to eppendorf tubes containing 10 μl of 37% formaldehyde and incubated for 8–10 mins at room temperature. Cells were spun down, washed with 1 ml of 80% ethanol and resuspended in 20 μl of 1 μg/ml DAPI before microscopy. Indirect immunofluorescence of meiotic spindles was carried out as previously described ( Bizzari & Marston, 2011).

Imaging of live cells at isolated timepoints was performed on ~1mm deep 2% agarose slides. In total, 100 μl of meiotic culture was spun down and resuspended in 5 μl of sporulation (SPO) media (0.3% Potassium acetate) and 2–3 μl of cell suspension was added to each agarose pad. The slide was covered with a glass coverslip and sealed with a molten mixture of vasoline:lamalin:paraffin (1:1:1) before microscopy.

Live-cell meiotic movies were generated using CellASIC® ONIX Y04D Microfluidics plates (Merck Millipore). All chambers on the plate were washed three times with 500 μl of SPO media before 200 μl of SPO media plus 1mM β-estradiol was added to chambers 1–6. Plates were then pre-incubated at 30°C 30 mins. After incubation, 200 μl of prophase I arrested cells (by Ndt80-depletion; Carlile & Amon, 2008) was loaded into chamber 8. A total of four different strains can be imaged on a single plate. The microfluidics plate was attached via a low-profile manifold to the CellASIC® ONIX Microfluidic Platform Control System, and the assembly was placed on a Deltavision Elite microscope. Cells were loaded, visualised, washed with β-estradiol-containing SPO media and imaged. 

Imaging of fixed cells or live cells at isolated time-points was carried out using a Zeiss Axio Imager Z1 and a Photometrics EMCCD camera. Images were taken using Micro-Manager v1.4 ( https://micro-manager.org/) and processed using ImageJ software v1.47 ( https://imagej.nih.gov/ij/). For the generation of microfluidics movies, a Deltavision® Elite live cell imaging system was utilised with an Olympus IX-71 microscope and a Photometrics EMCCD Cascade II camera. Multi-point images were taken using SoftWoRx v5.5 ( http://www.gelifesciences.com/), movies were assembled in Image-Pro Plus (Media Cybernetics) and processed in ImageJ v1.47.

### Quantification of fluorescence signal

Quantification of fluorescence signal was performed as described in Hoffman *et al*., 2001. In brief, the following equations were used:

FBk = (FO – Fi) * (Ai / (AO - Ai))

Fx = Fi - FBk

O and I represent outer and inner regions, respectively. The inner region (I) contained ~90% of the signal measured. The outer region (O) was at least twice the area of the inner region (Ai=16 pixels; AO=64 pixels), and was used to calculate the surrounding background (Bk) signal. F signifies integrated fluorescence signal, calculated from Raw Integrated Densities, and A is the area of the boxes. AO were 16 and 64 pixels, respectively.

### Electron microscopy

For sample preparation, 3 ml of culture from meiotic cell cycle time-course was vacuum filtered through a 0.45 μm Millipore filter. The cell paste was rapidly frozen under high pressure in a Wohlwend Compact 02 High Pressure Freezer. Frozen cell pellets were then freeze substituted in acetone containing 2% (w/v) osmium tetroxide and 0.1% (w/v) uranyl acetate at -80°C. Samples were slowly warmed to room temperature over three days. After washing cells twice in acetone, samples were embedded in Epon 812 resin (Hexion) through multiple changes of diluted resin with acetone (1:3, 1:1 and 3:1). Three more changes using undiluted Epon 812 resin were carried out over two days before resin was polymerised at 60–70°C overnight. Epon blocks were serially sectioned at a thickness of 70 nm and stained with 2% (w/v) uranyl acetate in sterile water for 8 mins, and then in Reynolds’ lead citrate for 3 mins. Sections were viewed on a Philips CM120 transmission electron microscope, and images were collected with a Gatan Orius CCD camera and processed using ImageJ v1.47.

### Immunoprecipitation

Meiotic cells were harvested and washed with sterile water by centrifugation at 4000 rpm for 6 mins. Cells were resuspended in 0.2x cell volume of sterile water before drop-freezing in liquid nitrogen. Cells were ground five times in a Retsch Mixer Mill MM400. For Cdc14-SZZ(TAP) purification, the yeast lysate was thawed in Hyman (50 mM Bis-Tris propane, pH7; 100 mM KCl; 5 mM EGTA; 10% (v/v Glycerol)) with inhibitors (5 μg/ml each chymostatin, leupeptin, antipain, pepstatin A, E-64; 4mM AEBSF (pefablock); 2mM benzamidine, 2mM PMSF, 0.4 mM LR-microcystin, N-ethylmaleimide (NEM), sodium orthovanadate, b-glycerolphosphate, sodium pyrophosphate). For Spc42-3FLAG purifications, we adapted SPB buffer ( Niepel *et al*., 2005) by addition of inhibitors as above. Following thawing, Triton X-100 was added to a final concentration of 1% (w/v) and samples were sonicated at 39% amplitude for 1 × 30 secs per 10 ml of lysate. Lysates were centrifuged at 4000 rpm for 5 mins at 4°C and the supernatant was transferred to a new 50 ml falcon tube. Immunoprecipitation was performed by adding 5 mg of rabbit IgG-coupled Dynabeads or 18 mg of M2 αFLAG-coupled Dynabeads per 30 g lysed yeast, and the lysates were rotated at 4°C for 2 h. Lysates were then washed five times in cold buffer without inhibitors and then transferred to a 1.5 ml eppendorf tube with 1 ml buffer. Residual buffer was removed, 25 μl of 1x NuPAGE® LDS sample buffer was added, samples were boiled at 100°C for 5 mins before 5 µl of β-mercaptoethanol was added and samples were boiled for a further 5 mins, spun down at 13000 rpm for 5 mins and loaded onto a precast NuPAGE® 8–12% Bis-Tris gel (Novex). Bands were visualized after staining using the Pierce silver staining kit (Thermo Scientific).

### Mass spectrometry

Protein bands were excised from Coomassie-stained NuPAGE® 8–12% Bis-Tris gels and washed alternatingly with 50 mM ammonium bicarbonate and acetonitrile solutions until Coomassie staining was removed. Gel pieces were treated with 10 mM DTT in 50 mM ammonium bicarbonate for 30 mins at 37°C, then DTT was removed and samples were washed with acetonitrile. A total of 55 mM iodoacetamide in 50 mM ammonium bicarbonate was added to the gel slices, and these were incubated at room temperature in the dark for 20 mins. After washing again with 50 mM ammounium bicarbonate and acetonitrile, gel pieces were incubated with trypsin for 15 mins on ice, and then samples were transferred to 37°C for overnight digestion. The following morning, digestion reactions were treated with 0.1% (w/v) trifluoroacetic acid and left for 15 mins to allow peptides to diffuse from the gel. Samples were then passed through an equilibrated StageTip consisting of two layers of Empore Disks C18 within a pipette tip ( Rappsilber *et al.,* 2007). A single StageTip was used per sample, as peptides within samples bind to StageTips. Peptides were later eluted for analysis via mass spectrometry (MS), performed as previously described ( Sarangapani *et al*., 2014). MS data was compiled in MaxQuant v1.4.1.2 ( http://www.coxdocs.org/doku.php?id=:maxquant:start). Quantitative analysis was performed using Perseus v1.5.1.6 ( http://www.coxdocs.org/doku.php?id=perseus:start). MaxQuant LFQ intensities were normalized to Spc42-FLAG, filtered to remove contaminants, data was logarithmised to log2(x), then further filtered to only include proteins present in 5 out of the 6 groups (i.e. 2 wild type and 3 *cdc14-1*). The statistical test for significance was a t-test (FDR = 0.05, s0=1). No changes were found to reach significance.

## Results

### Spindle disassembly is only moderately delayed in *cdc14-1* mutants

A hallmark of mitotic exit is spindle disassembly, an event that is critically dependent on Cdc14 in budding yeast mitosis ( Stegmeier & Amon, 2004). Initial analysis of fixed temperature-sensitive *cdc14-1* mutant cells undergoing meiosis at the restrictive temperature revealed an increased frequency of cells with long spindles characteristic of anaphase I, suggesting blocked spindle disassembly and impaired meiosis I exit ( Marston *et al*., 2003). However, live-cell imaging revealed that meiosis I spindles frequently disassemble in *cdc14-1* cells, only to reassemble at the presumptive time of meiosis II ( Bizzari & Marston, 2011), suggesting that Cdc14 may be refractory for spindle disassembly and meiosis I exit.

**Figure 1.**
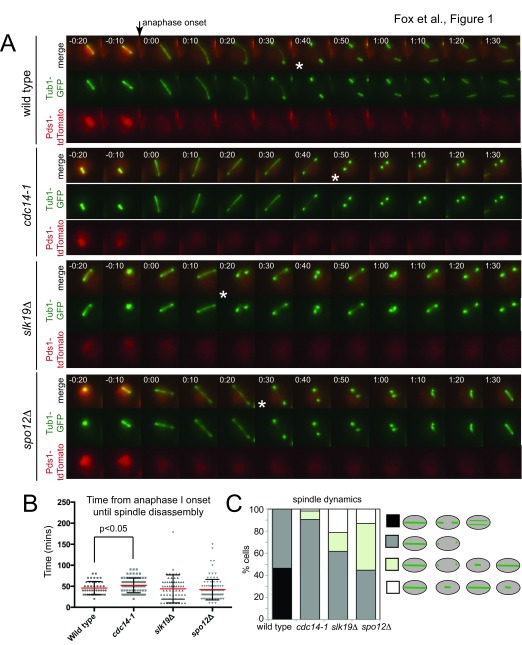
Meiosis I spindle disassembly in the absence of functional Cdc14. Wild type (AM16065), *cdc14-1* (AM16066), *slk19Δ* (AM16110) and *spo12Δ* (AM15985) cells carrying *TUB1-GFP* and *PDS1-tdTomato* were induced to sporulate, released from prophase I arrest and imaged at 10 min intervals for a total of 12 h in a microfluidics device. ( **A**) Representative images are shown. The black arrow denotes Pds1 degradation, marking entry of cells into anaphase I. The white asterisk marks the time of spindle breakdown. ( **B**) The time taken for complete spindle disassembly after Pds1 degradation was recorded for individual cells and plotted for a total of 67 wild type cells and 100 of each of *cdc14-1*, *slk19Δ* and *spo12Δ* cells. Mean rates of spindle breakdown are shown (red line), with error bars representing standard deviation. The two-tailed Student’s *t*-test was used to calculate p values. ( **C**) Inactivation of Cdc14 results in abnormal spindle behaviour. Cells were categorised based on spindle morphology as indicated in the legend.

To establish the importance of Cdc14 in spindle disassembly following meiosis I, we determined the time from anaphase I onset until spindle breakdown in live cells with impaired Cdc14 function. Securin (Pds1-tdTomato) degradation was used a marker for anaphase I onset and the time taken for the meiosis I spindle (GFP-Tubulin) to completely disassemble after Pds1 proteolysis was measured in individual cells. In the wild type example ( Figure 1A), spindle disassembly was observed 40 min after anaphase I onset, after which meiosis II spindles formed (note that Pds1-tdTomato is not visualised in meiosis II cells, presumably due to slow maturation of the fluorophore ( Matos *et al*., 2008)). Spindle disassembly occurred 45.7 min after anaphase I, on average ( Figure 1B) and was observed in 100% of wild type cells ( Figure 1C). In ~82% *cdc14-1* mutant cells, anaphase I spindles broke down and a new spindle did not assemble ( Figures 1A and C), which is consistent with what we previously reported ( Bizzari & Marston, 2011). We observed a modest, yet significant, increase (up to 52.2 min) in the time from anaphase onset to spindle disassembly in *cdc14-1* cells ( Figure 1B). In contrast, *slk19Δ* and *spo12Δ* cells, which retain Cdc14 in the nucleolus during meiosis I ( Buonomo *et al*., 2000; Marston *et al*., 2003), disassembled anaphase I spindles with a timing comparable to wild type cells (43.8 and 41.9 min, respectively; Figures 1A and 1B). As reported previously for *cdc14-1* mutants ( Bizzari & Marston, 2011), spindle reassembly at the presumptive time of meiosis II was observed in a fraction of *slk19Δ* and *spo12Δ* cells, though the extent to which this occurred varied between the different mutants for reasons that are unclear. Taken together, these findings indicate that, while FEAR and Cdc14 appear to work together to ensure that two spindles are produced during meiosis II, Cdc14 may promote timely meiosis I spindle disassembly through a FEAR-independent mechanism. Nevertheless, spindle disassembly invariably occurs in *cdc14-1* mutants, raising the possibility that, in contrast to the critical requirement for Cdc14 for exit from mitosis, Cdc14 is not absolutely required for CDK down-regulation at meiosis I exit. Consistently, degradation of the major meiosis I cyclin Clb1 is not obviously delayed in *cdc14-1* mutants ( Bizzari & Marston, 2011). Similarly, a recent study observed timely meiosis II spindle disassembly following inactivation of Cdc14 using the distinct *cdc14-3* temperature-sensitive allele ( Argüello-Miranda *et al.*, 2017). Although we cannot completely rule out retention of partial activity by the temperature sensitive Cdc14 proteins, together these findings suggest that Cdc14 is more critical for spindle disassembly in mitosis than meiosis.

### Cdc14 associates with the SPB at the meiosis I to meiosis II transition

**Figure 2.**
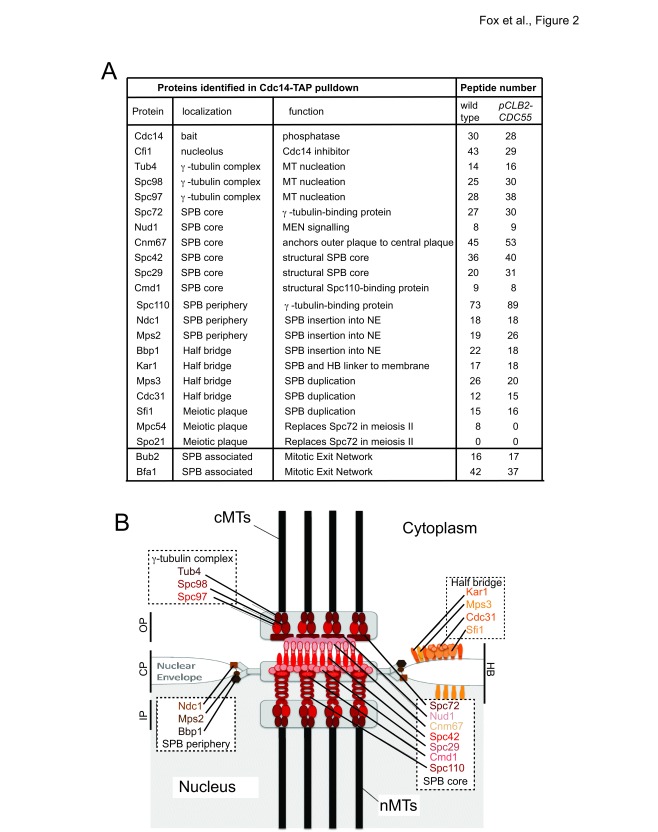
The centrosome/SPB co-purifies with Cdc14 during meiosis. ( **A**) Wild type (AM9434) and *pCLB2-3HA-CDC55* (AM9319) cells carrying *CDC14-SZZ* were harvested 4 h after induction of sporulation. Cdc14-SZZ-associated complexes, purified on IgG-coupled beads, were analysed by mass spectrometry. The number of identified peptides of the indicated proteins is given. See also https://osf.io/g5cmh/ ( Marston, 2016). ( **B**) Schematic diagram of the budding yeast spindle pole body.

We took an unbiased approach to identify cellular processes targeted by Cdc14 to regulate the meiosis I to meiosis II transition. Following its release from the nucleolus during anaphase I, Cdc14 is expected to associate with, and dephosphorylate, substrates that facilitate the transition to meiosis II. We reasoned that identification of Cdc14 interacting partners in both wild type cells and *pCLB2-CDC55* cells, in which Cdc14 is ectopically released from the nucleolus, would inform on the processes it regulates. Anti-FLAG immunoprecipitates from wild type and *pCLB2-3HA-CDC55* cells harvested 4 h after induction of meiosis and carrying *CDC14-3FLAG* were analysed by mass spectrometry. Despite similar Cdc14 peptide counts in wild type and *pCLB2-3HA-CDC55* samples, we observed a lower Cfi1/Net1 peptide count in the latter sample, consistent with premature release of Cdc14 from the nucleolus in Cdc55-deficient cells ( Figure 2A). Interestingly, however, the predominant class of proteins identified in both samples were components of the yeast centrosome/spindle pole body (SPB) ( Figures 2A and B).

**Figure 3.**
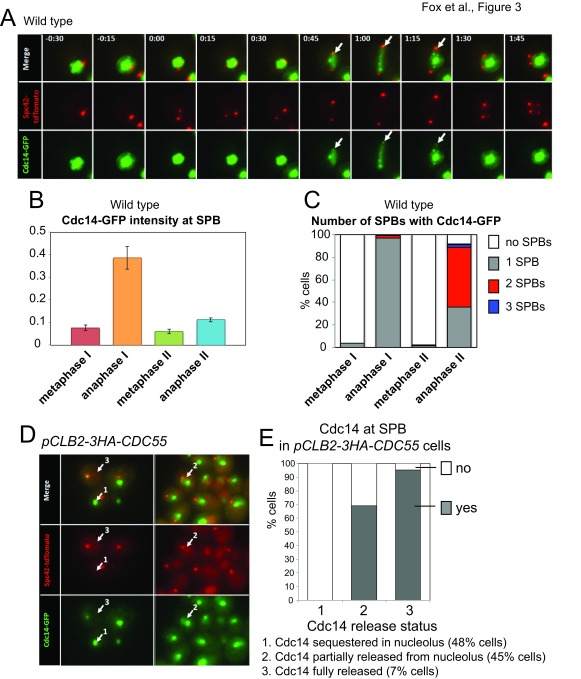
Cdc14 localizes asymmetrically at SPBs during anaphase I. ( **A**) Sporulating *CDC14-GFP SPC42-tdTomato* cells (AM11517) were imaged in a microfluidics chamber at 15 min intervals for a total of 12 h. Example of a cell in which Cdc14-GFP localizes asymmetrically to one SPB during anaphase I (arrows). ( **B** and **C**) Wild type cells as in ( **A**) were released from a prophase I arrest and imaged at 30 min intervals on agarose pads. ( **B**) The ratio of Cdc14-GFP/Spc42-tdTomato signal per SPB is shown with error bars representing standard error. Cells were classified into different cell cycle stages (metaphase I, anaphase I, metaphase II, anaphase II) by scoring the number of SPBs, the distance between them and Cdc14 nucleolar sequestration. The two-tailed Student’s *t-*test was used to calculate p values (* p<0.001) *n=*50 cells. ( **C**) Co-localization of Cdc14-GFP with SPBs was scored in the indicated cell cycle stages. ( **D** and **E**) Cdc14 localises prematurely to SPBs in the absence of Cdc55. *pCLB2-3HA-CDC55* cells carrying *CDC14-GFP* and *SPC42-tdTomato* (AM13123) were induced to sporulate, released from *pGAL-NDT80* block and imaged 3h later on agarose pads. Cdc14 localisation was classified into three categories: sequestered in nucleolus (1), partially released from nucleolus (2) and completely released from nucleolus (3). ( **D**) Example images of with numbered arrows showing examples of each category. ( **E**) Co-localisation of Cdc14 with SPBs was scored in 100 cells of each category.

To determine the timing of Cdc14 association with the SPB during meiosis, we imaged live cells carrying *CDC14-GFP* and the SPB marker, *SPC42-tdTomato* undergoing meiosis. As previously reported, in wild type cells, Cdc14 is sequestered in the nucleolus throughout prophase I and metaphase I of meiosis and, accordingly, we did not observe co-localization with SPBs at these stages (( Buonomo *et al*., 2003; Marston *et al*., 2003; Matos *et al*., 2008); Figure 3A). During anaphase I, however, concomitant with its release from the nucleolus, Cdc14-GFP was detected at the SPB ( Figure 3A, arrows). To confirm the timing of Cdc14 association with the SPB we determined the ratio of intensity of Cdc14-GFP and Spc42-tdTomato fluorescence ( Figure 3B). This revealed the strongest association of Cdc14-GFP with the SPB in anaphase I, with a weaker association in anaphase II ( Figure 3B). Interestingly, Cdc14-GFP localized asymmetrically, generally associating with just one of the two SPBs in anaphase I, or two of the four SPBs during anaphase II, with no detectable SPB association during metaphase I or metaphase II ( Figure 3C).

We, and others, previously showed that ectopic release of Cdc14 prevents nuclear division in *pCLB2-3HA-CDC55* cells ( Bizzari & Marston, 2011; Kerr *et al*., 2011). To determine whether premature association of Cdc14 with the SPB could underlie this phenotype, we induced *pCLB2-3HA-CDC55* cells carrying *CDC14-GFP* and *SPC42-tdTomato* to undergo meiosis and categorised cells based on the localization of Cdc14-GFP: nucleolar sequestration (class 1); partial release (class 2) or complete release (class 3) ( Figures 3D and E). Cdc14-GFP was detected at the SPB in virtually all *pCLB2-3HA-CDC55* cells where Cdc14-GFP was either completely or partially released from the nucleolus ( Figure 3E). Note that the vast majority of *pCLB2-3HA-CDC55* cells contain only a single Spc42-tdTomato foci, therefore it was not possible to address whether Cdc14-GFP remains asymmetric in these cells.

**Figure 4.**
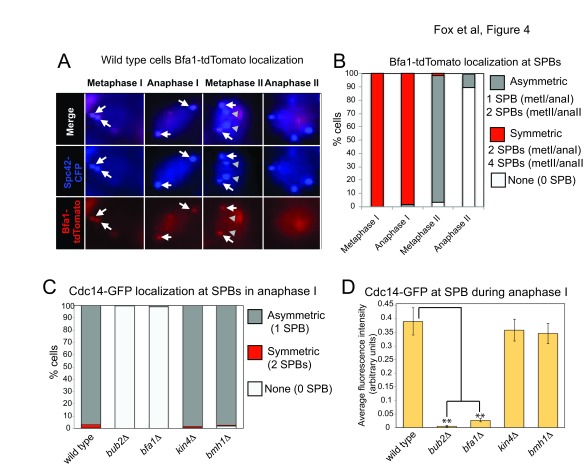
Asymmetric localisation of Cdc14 to SPBs is Bfa1/Bub2-dependent. ( **A** and **B**) Bfa1 localises symmetrically to SPBs during meiosis I. Wild type (AM17740) cells containing *SPC42-CFP, BFA1-tdTomato and CDC14-GFP* were induced to sporulate, released from *pGAL-NDT80* block and imaged at 30 minute intervals on agarose pads. Cells were classified into different meiotic stages based on number of SPB foci, distance between SPBs and Cdc14 nucleolar sequestration. ( **A**) Representative images of Bfa1 localisation. White arrows indicate Bfa1-tdTomato co-localization with Spc42-CFP foci. Grey arrowheads indicate Spc42-CFP foci where Bfa1-tdTomato is absent. ( **B**) Co-localisation of Bfa1 with SPBs was scored throughout meiosis in 200 cells at each stage. Note that Spc42-CFP bleeds through to the GFP emissions channel so Cdc14-GFP signal is not shown, though its nucleolar sequestration and release was used to classify meiotic stages. ( **C** and **D**). Wild type (AM11517), *bfa1Δ* (AM16079), *bub2Δ* (AM15543), *kin4Δ* (AM17134) and *bmh1Δ* (AM17341) cells containing *CDC14-GFP* and *SPC42-tdTomato* were induced to sporulate, released from a *pGAL-NDT80* block and imaged at 30 minute intervals on agarose pads. ( **C**) Co-localisation of Cdc14 with SPBs was scored in 100 anaphase I cells. ( **D**) The ratio of Cdc14-GFP/Spc42-tdTomato signal per SPB per cell was quantified, with error bars representing standard error. Cells were classified as anaphase I based on distance between SPB foci and Cdc14 release. The two-tailed Student’s *t-*test was used to calculate significance. ** indicates p<0.001. n = 100 foci (50 cells).

We sought to identify factors that are required for Cdc14 localization to SPBs during anaphase I. During mitosis, components of the MEN are associated with the SPB. However, the MEN is dispensable for the meiosis I to meiosis II transition and the majority of its components are not found at SPBs ( Attner & Amon, 2012; Kamieniecki *et al*., 2005; Pablo-Hernando *et al*., 2007). An exception is the two component GAP, Bub2/Bfa1 which localizes symmetrically at SPBs during metaphase I, anaphase I but asymmetrically during metaphase II ( Attner & Amon, 2012; Figures 4A and B). We found that Cdc14-GFP association with SPBs was abolished in both *bub2Δ* and *bfa1Δ* anaphase I cells ( Figures 4C and D). We conclude that upon release from the nucleolus in anaphase I and anaphase II, Cdc14 associates asymmetrically at SPBs in a manner dependent on Bub2/Bfa1.

### Generation of 4 Spc42-tdTomato foci during meiosis II depends on Cdc14

**Figure 5.**
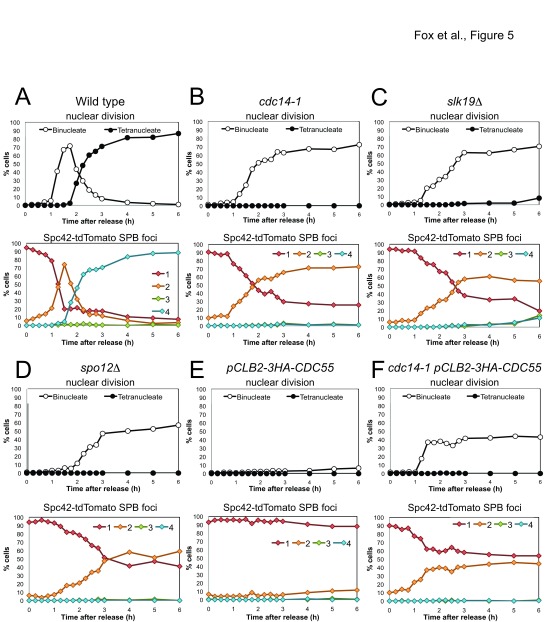
Active Cdc14 is required for cells to form four distinct Spc42-tdTomato foci in meiosis. Wild type (AM13989; **A**), *cdc14-1* (AM16163; **B**), *cdc55mn* (AM15984; **C**), *slk19Δ* (AM16020; **D**) s *po12Δ* (AM16064; **E**) and *cdc14-1 cdc55mn* (AM17904; **F**) cells carrying *SPC42-tdTomato* were induced to sporulate and released from a *pGAL-NDT80* block. The percentages of binucleate and tetranucleate cells (upper graph) and the number of Spc42-tdTomato per cell were scored in 200 cells at each timepoint (lower graph).

**Figure 6.**
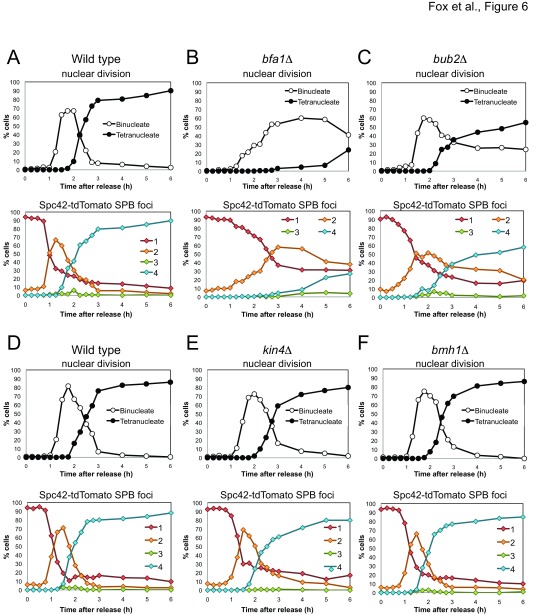
Bfa1 and Bub2 are required for timely re-duplication of SPBs in meiosis. ( **A**– **C**) Wild type (AM13989; **A**), *bfa1Δ* (AM16019; **B**) and *bub2Δ* (AM16080; **C**) cells containing *SPC42-tdTomato* were induced to sporulate and released from *pGAL-NDT80* block. The percentages of binucleate and tetranucleate cells (upper graph) and the number of Spc42-tdTomato per cell were scored in 200 cells at each timepoint (lower graph). ( **D**– **F**) Wild type (AM13989; **D**), *kin4Δ* (AM17134; **E**) and *bmh1Δ* (AM17341; **F**) cells containing *CDC14-GFP* and *SPC42-tdTomato* were induced to sporulate, released from a *pGAL-NDT80* block and imaged at 30 min intervals on agarose pads. The percentages of binucleate and tetranucleate cells (upper graph) and the number of Spc42-tdTomato per cell were scored in 200 cells at each timepoint (lower graph).

Recently, Cdc14 has been identified as a licensing factor that enables SPB duplication upon exit from mitosis ( Avena *et al*., 2014; Elserafy *et al*., 2014). Taken together with our findings above, this suggests that a major function of Cdc14 during meiosis could be to license a second round of SPB duplication, thereby enabling the assembly of two spindles in meiosis II. To test this idea, we monitored SPB number in wild type cells or where Cdc14 was inactivated ( *cdc14-1*) by scoring Spc42-tdTomato foci as cells progressed synchronously through meiosis after release from a prophase I block ( Figures 5A and B). As expected, wild type cells produced two, then four Spc42-tdTomato foci concomitant with the appearance of binucleate and tetranucleate cells ( Figure 5A). In contrast, and consistent with the observed single nuclear division, *cdc14-1* cells produced a maximum of two Spc42-tdTomato foci ( Figure 5B), as did cells lacking the two FEAR activators, Spo12 and Slk19 ( Figures 5C and D). Upon depletion of Cdc55, a single Spc42-tdTomato focus was observed in the majority of cells ( Figure 5E), indicating a failure in the first round of SPB duplication or separation. Furthermore, this lack of SPB duplication/separation prior to meiosis I in Cdc55-depleted cells was a consequence of ectopic Cdc14 activation, since *cdc14-1 pCLB2-3HA-CDC55* cells produced two Spc42-tdTomato foci, similar to *cdc14-1* single mutant cells ( Figure 5F). Consistent with a requirement for Cdc14 at SPBs, a large fraction of *bub2Δ* and *bfa1Δ* cells completed only a single meiotic division with only 2 Spc42-tdTomato foci ( Figures 6A–C). During mitosis, Bub2/Bfa1 is asymmetrically localized on SPBs, but this asymmetry is broken in response to defective spindle positioning, in a manner dependent on Kin4 and Bmh1 ( Gryaznova *et al*., 2016; Maekawa *et al*., 2007; Monje-Casas & Amon, 2009). We found, however that Kin4 and Bmh1 are dispensable for either the asymmetric SPB localization of Cdc14 ( Figure 4D) or the execution of two meiotic divisions ( Figures 6E–F), at least under normal conditions. These findings indicate that localization of Cdc14 at the SPB is important for the successful execution of the meiosis I to meiosis II transition.

### Cdc14 is essential for SPB duplication at the meiosis I to meiosis II transition

**Figure 7.**
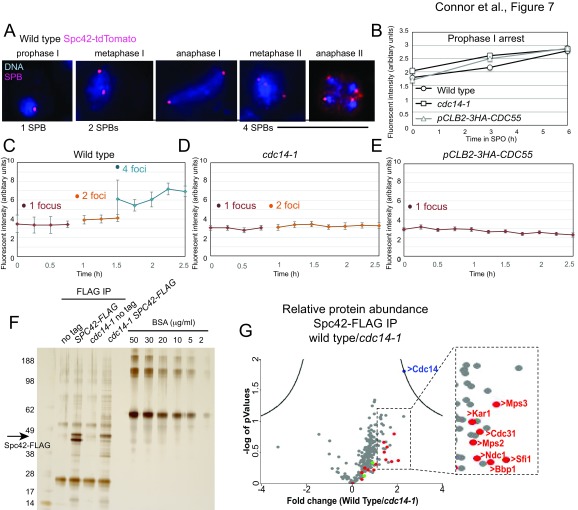
Quantitative analysis of SPBs in meiosis indicates a requirement for Cdc14 in SPB duplication. ( **A**) Wild type (AM13989) cells containing *SPC42-tdTomato* were induced to sporulate, released from *pGAL-NDT80* block and imaged at 15 minute intervals for a total of 12 hours in a microfluidics chamber. Representative images of wild type cells are shown. ( **B**) SPBs are duplicated at meiotic prophase. Wild type *cdc14-1* (AM16163), and *pCLB2-3HA-CDC55* (AM15984) cells containing *SPC42-tdTomato* and *pGAL-NDT80* and were resuspended in sporulation medium in the absence of β-oestradiol and imaged immediately (t=0) or after 3 and 6h as they progress into the prophase I arrest due to the absence of Ndt80. Individual SPB foci were quantified and mean total SPB fluorescence intensity (F _i_) per cell was plotted, with error bars (obscured by the markers) representing standard error, n=100 per timepoint. ( **C**– **E**) SPB fluorescence was quantified as in B from movies of live cells after release from the prophase I arrest by addition of β-oestradiol as in ( **A**). The first time point at which a cell contained 2 Spc42-tdTomato foci was defined as 1h and SPB fluorescence in the preceding 4 (1 focus) and following 4 time points was quantified (n=10 cells). Note that at the 1.5h time point 7 wild type cells carried 2 Spc42-tdTomato foci, while 3 wild type cells carried 4 Spc42-tdTomato foci. ( **F** and **G**) LFQ Proteomic analysis of SPB composition and environment in wild type and *cdc14-1* cells. Strains used were AM1835 (no tag), AM11444 ( *SPC42-3FLAG*), AM9459 ( *cdc14-1*) and AM11443 ( *cdc14-1 SPC42-3FLAG*) After 4 h, cells were harvested and SPBs purified by anti-FLAG immunoprecipitation. Peptides were generated by in-gel trypsin digestion and LC-MS data sets for 3 biological replicas was analysed using MaxQuant software. ( **F**) Example silver stained SDS-PAGE showing Spc42-3FLAG immunoprecipitates of one of three biological replicas used in LFQ proteomic analysis. BSA standards were used to estimate protein concentration. ( **G**) Statistical analysis of relative LFQ intensity output was carried out using Perseus. Volcano plot shows –log of P values versus ratio of wild type/c *dc14-1* for all 254 proteins in >5 columns. No significant change in composition and environment was observed between wild type and *cdc14-1* SPBs. (FDR = 0.05, s0 = 1). Proteins of interest are highlighted as follows: red = SPB components; blue = Cdc14; green = Bfa1/Bub2. See also https://osf.io/g5cmh/ ( Marston, 2016).

To determine whether *cdc14-1* and *pCLB2-3HA-CDC55* mutants are defective in SPB duplication or separation, we initially used quantitative fluorescence microscopy. We measured the total intensity of the central SPB component Spc42-tdTomato within each cell ( Figure 7A). First, we examined cells progressing from meiotic entry (induced by resuspension in sporulation medium) into a prophase I block (by preventing *NDT80* expression) ( Carlile & Amon, 2008). At meiotic entry, the majority of cells are in G1 phase of the cell cycle and expected to contain 1 SPB with a full bridge that is already licensed/competent for assembly of a new SPB alongside it. In meiosis, separation of these SPBs occurs only at prophase I exit. Consistent with this idea, we observed an approximately 1.5 fold increase in Spc42-tdTomato intensity as wild type cells progressed from G1 into the prophase I arrest ( Figure 7B). Upon Cdc14 inactivation ( *cdc14-1*) or Cdc55 depletion ( *pCLB2-3HA-CDC55*) at meiotic entry we observed a similar increase in Spc42-tdTomato intensity in prophase I, suggesting that Cdc14 and Cdc55 are not required for the assembly of a new SPB, once licensing has occurred ( Figure 7B). Next we examined the intensity of Spc42-tdTomato as cells progressed from the prophase I block into the meiotic divisions ( Figures 7C–E). In wild type cells, overall Spc42-tdTomato intensity was slightly increased in the cells with 2 Spc42-tdTomato foci, compared to the cells with 1 Spc42-tdTomato focus suggesting that SPB re-duplication had occurred between meiosis I and meiosis II, though interestingly the increase was much less than twofold (~x1.18) ( Figure 7C). Spc42-tdTomato intensity was greatly increased in cells with 4 Spc42-tdTomato foci, perhaps in preparation for SPB maturation that occurs in meiosis II and which is important for spore formation ( Neiman, 2011). In *cdc14-1* cells, the overall intensity of Spc42-tdTomato foci did not increase, even though cells produced 2 Spc42-tdTomato foci (x1.07) ( Figure 7D), suggesting that SPB re-duplication failed to occur prior to SPB separation. The single Spc42-tdTomato focus of *pCLB2-3HA-CDC55* cells also did not increase in Spc42-tdTomato intensity throughout the timecourse (x0.98), suggesting that both SPB separation and re-duplication fail to occur upon Cdc55 depletion.

To gain further insight into how Cdc14 influences SPB morphogenesis during meiosis, we compared the composition of the SPB in wild type and *cdc14-1* cells by quantitative mass spectrometry. SPBs were purified from wild type and *cdc14-1* cells undergoing meiosis and carrying *SPC42-3FLAG* by immunoprecipitation using anti-FLAG antibodies ( Figure 7F). Comparison of relative peptide intensities for three biological replicates ( Figure 7G) indicated significant depletion of Cdc14 on SPBs from *cdc14-1* cells, consistent with the idea that the mutant protein fails to associate with the SPBs. Though not reaching the stringent cut off for statistical significance (FDR=0.05), we further noticed that components of the SPB half bridge tended to be depleted on *cdc14-1* SPBs. Interestingly, the half bridge component, Sfi1, which was recently confirmed as a Cdc14 target in mitosis ( Avena *et al*., 2014; Elserafy *et al*., 2014) showed the greatest change in abundance. These results are consistent with the idea that a major function of Cdc14 at the meiosis I to meiosis II transition is to enable half bridge extension, thereby allowing SPB re-duplication.

**Figure 8.**
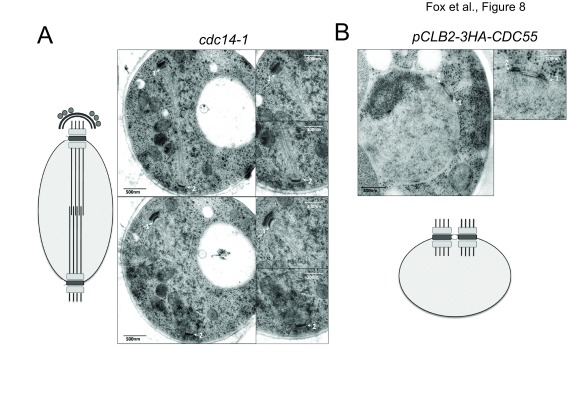
*cdc14-1* and *pCLB2-3HA-CDC55* mutants arrest with unduplicated and unseparated SPBs, respectively. c *dc14-1* (AM16077) and *pCLB2-3HA-CDC55* (AM16198) cells were induced to sporulate, released from *pGAL-NDT80* block and serial sections were analysed by electron microscopy. ( **A**) Two sections of the same representative *cdc14-1* cell containing a long bipolar spindle with two unduplicated SPBs (arrows 1,2) embedded in the nuclear envelope are shown. Right panels show higher magnification of SPBs in the same cell. Model illustrates in dark grey the cellular structures visible in EM images. Note that Pro-spore wall formation is observed around SPB1 and secretory vesicle recruitment is evident. ( **B**) Representative *pCLB2-3HA-CDC55* cell containing two SPBs connected via a bridge structure. See also 10.17605/OSF.IO/G5CMH ( Marston, 2016).

To examine SPB morphogenesis more directly, we analyzed *cdc14-1* and *pCLB2-3HA-CDC55* meiotic cells by electron microscopy. As predicted by the quantitative fluorescence microscopy, SPB re-duplication was not observed in *cdc14-1* mutants (n=8) and cells arrested with two unduplicated SPBs. We observed late meiosis II events in 3/8 cells. In the example shown ( Figure 8A) a long spindle connects two unduplicated SPBs. Assembly of the outer plaque and vesicles are apparent at one of the SPBs (SPB 1, white arrow), indicating that the cell is in a late stage of meiosis II, though outer plaque formation has not been initiated at the other SPB (SPB 2, white arrow). In *pCLB2-3HA-CDC55* cells, also as predicted from our quantitative fluorescence microscopy, two side-by-side SPBs connected by a half bridge were invariably observed ( *n=6*; Figure 8B). However, we found no evidence of over-duplication of *SPBs* in *pCLB2-3HA-CDC55* cells. This suggests that Cdc14 must be held inactive during early meiosis to allow SPB separation and acts in a licensing step, rather than as an assembly factor.

## Discussion

**Figure 9.**
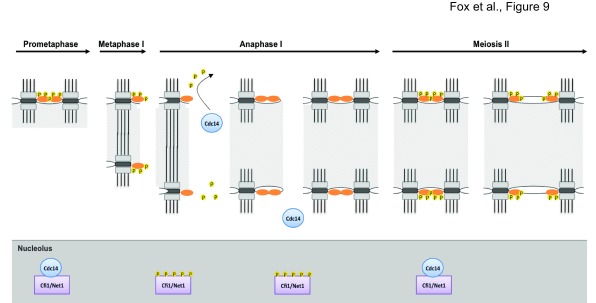
Model for Cdc14 regulation of SPB duplication during meiosis. Two duplicated SPBs are attached to one another in prometaphase by a full-bridge structure consisting primarily of dimerised Sfi1 (orange), an interaction stabilised by Cdc31. Cdc14 is sequestered in the nucleolus, preventing the dephosphorylation of Sfi1 and possibly other SPB components. Sfi1 phosphorylation (yellow) results in full-bridge cleavage, enabling the separation of SPBs in metaphase I. In anaphase I, release of Cdc14 from inhibition results in Sfi1 dephosphorylation. Sfi1 dimerisation occurs and two half-bridge structures elongate, initiating SPB re-duplication. When Cdc14 is re-sequestered at meiosis I exit, the full-bridges are severed, resulting in the separation of four distinct SPBs in meiosis II.

The existence of two consecutive rounds of chromosome segregation without an intervening S phase is a characteristic feature of meiosis that underlies sexual reproduction. Unique, yet poorly understood, controls allow a second round of spindle formation, but prevent a second round of DNA replication. Our results implicate Cdc14 regulation as being central to this distinction. In mitosis, following chromosome segregation, MEN-dependent release of Cdc14 triggers CDK inactivation permitting both the re-licensing of SPBs and DNA replication origins. In contrast, following meiosis I, MEN is not active ( Attner & Amon, 2012) and Cdc14 release is under the control of only the FEAR network ( Buonomo *et al*., 2003; Kamieniecki *et al*., 2005; Marston *et al*., 2003), which is incapable of triggering mitotic exit ( Yellman & Roeder, 2015). Here we show that FEAR-dependent Cdc14 is critical to initiate SPB duplication, thereby enabling assembly of two separate meiosis II spindles. We show that Cdc14 released by the FEAR network associates with the SPB in a Bub2/Bfa1-dependent manner and provide evidence that SPB-localized Cdc14 is critical to trigger SPB duplication. Based on recent findings in mitotic cells ( Avena *et al*., 2014; Elserafy *et al*., 2014), we suggest that Cdc14 re-licenses SPBs through dephosphorylation of half-bridge components, in particular Sfi1 during anaphase I ( Figure 9). Overall, our findings show that Cdc14 is required to re-license SPB duplication between meiosis I and meiosis II and that its retention in the nucleolus during early meiosis is required to allow SPB separation during meiosis I.

### SPB localization of Cdc14

Bub2/Bfa1-dependent association of Cdc14 with the SPB is not unique to meiosis, indeed it has been observed in early mitosis ( Pereira *et al*., 2002a; Yoshida *et al*., 2002), suggesting that it is a FEAR-triggered event here too. SPB-localized Cdc14 has been implicated both in MEN activation in early anaphase and, through Bfa1 dephosphorylation, in MEN inactivation in late anaphase ( Pereira *et al*., 2002b). An attractive possibility, which remains to be tested, is that in meiosis I, Cdc14 at the SPB acts to maintain Bfa1/Bub2 in the dephosphorylated state, thereby preventing MEN activation. Our data also suggest another function for SPB-localized Cdc14 during meiosis I, to trigger SPB duplication.

Curiously, we observed Cdc14 at a single SPB during anaphase I. Asymmetric localization of Cdc14 and MEN components is observed in budding yeast mitosis, where the requirement to partition the nucleus through the bud neck imposes an intrinsic polarity on cell division. The asymmetric localization of MEN components, both on the SPB and within the bud contributes to the spindle position checkpoint which prevents mitotic exit in response to spindle alignment defects ( Caydasi & Pereira, 2012). However, during meiosis I, MEN is not active ( Attner & Amon, 2012) and meiotic yeast cells are not obviously polarized. Furthermore, Cdc14 activity is presumably required at both SPBs during anaphase I to trigger their duplication, thereby ensuring production of a pair of spindles in meiosis II. While Cdc14 is detectable by microscopy only on 1 SPB during anaphase I, we speculate that undetectable levels of Cdc14 on the other SPB are sufficient to trigger SPB duplication. This however, raises the question of how and why Cdc14 is more concentrated on a single SPB, particularly considering that Bub2/Bfa1 is itself symmetrically localized. The origin and significance of the asymmetric localization of Cdc14 at the SPB during anaphase I therefore remain unexplained.

### Control of the cell cycle at meiosis I exit

Although Cdc14 is essential for mitotic exit, accumulating evidence suggests that Cdc14 plays a lesser role in CDK down-regulation at meiosis I exit. We found that spindle assembly is only slightly delayed in cells with impaired Cdc14 activity and cyclin destruction appears to occur on schedule in *cdc14-1* cells ( Bizzari & Marston, 2011; Kerr *et al*., 2011; Tibbles *et al*., 2013). Instead it is likely that cyclin degradation upon APC ^Cdc20^ activation at anaphase I onset initiates meiosis I exit. Understanding how this is regulated to ensure step-by-step release of cohesion, spindle elongation and spindle disassembly at meiosis I is an important priority for the future.

## Data availability

Source data for mass spectrometry results from Figures 2A (Fox_Data 1) and 7G (Fox_Data 2), example movies of spindle elongation ( Figure 1), additional examples of electron microscopy images, source data files for quantification of SPB fluorescence ( Figure 7) and source data files for quantification of Cdc14-GFP localization at SPBs ( Figure 3 and Figure 4) are available at: https://osf.io/g5cmh/ (doi, 10.17605/OSF.IO/G5CMH; Marston, 2016).
