# Supplementary material for: Cdc14 phosphatase directs centrosome re-duplication at the meiosis I to meiosis II transition in budding yeast
Source: Wellcome Open Res. 2017 Feb 21;2:2. Originally published 2017 Jan 5. [Version 2] doi: 10.12688/wellcomeopenres.10507.2 (PMC5266553; doi:10.12688/wellcomeopenres.10507.2)
Supplement: Peer review history

# Peer review: Referee response for version 2

**DOI:** 10.21956/wellcomeopenres.11794.r20436

**Published:** 2017-03-02

**Authors:** Simonetta Piatti

**Affiliations:** 1 Centre de Recherche en Biologie Cellulaire de Montpellier (CRBM), Montpellier, France

Authors have addressed all my comments and suggestions. Altogether it is a nice piece of work, carefully designed and executed.

I have read this submission. I believe that I have an appropriate level of expertise to confirm that it is of an acceptable scientific standard.

# Peer review: Referee response for version 2

**DOI:** 10.21956/wellcomeopenres.11794.r20434

**Published:** 2017-02-24

**Authors:** Soni Lacefield

**Affiliations:** 1 Department of Biology, Indiana University Bloomington, Bloomington, IN, USA

The authors have addressed my concerns and made appropriate changes to their manuscript in their revision.

I have read this submission. I believe that I have an appropriate level of expertise to confirm that it is of an acceptable scientific standard.

# Peer review: Referee response for version 1

**DOI:** 10.21956/wellcomeopenres.11325.r19028

**Published:** 2017-01-24

**Authors:** Kayoko Tanaka

**Affiliations:** 1 Department of Molecular and Cell Biology, University of Leicester, Leicester, UK

The centrosome is a fascinating organelle; it serves as a scaffolding platform for numerous cell cycle regulators in addition to the major microtubule organising centre, which plays an essential role in chromosome segregation by acting as the spindle pole. During the mitotic cell cycle, a rigorous licensing mechanism that involves phosphorylation and dephosphorylation of the centrosome components restricts the number of centrosome duplications to exactly one per cell cycle. Physiological importance of this regulation is underlined by the fact that tumour cells are often associated with abnormally amplified centrosomes. The timing of the centrosome duplication in many organisms coincides with S phase, where DNA replication takes place. Common cell cycle regulators, such as CDKs, trigger both centrosome duplication and DNA replication events.

 In meiosis, however, centrosome duplication must be uncoupled from DNA replication, because, no DNA replication must occur at the transition from meiosis I (MI) to meiosis II (MII). Although the mechanism of uncoupling of the centrosomal and DNA replication cycles in meiosis is mostly unclear, studies in *Xenopus* oocytes ^1^ and the fission yeast ^2^ found significant residual CDK activities at MI - MII transition, suggesting that these CDK activities may inhibit DNA replication, hence, decoupling the centrosome cycle from the DNA replication cycle. However, further supporting evidence for it or the molecular basis that directs the meiotic centrosome cycle has still been missing.

 In this manuscript, Fox *et al* ^3^ used the budding yeast as a model and showed that Cdc14 phosphatase is one of the essential regulators of the meiotic centrosome duplication. By exploiting a conditional *cdc14-1* mutant, an absolute requirement of Cdc14 function in meiotic centrosome duplication was clearly demonstrated through extensive live and fixed cell imaging of the cells undergoing MI-MII synchronously. Observation using electron microscopy provided further supporting evidence.  

 The manuscript is very well written, the quality of the data (which are mostly very clearly presented) is high and sound data interpretations have been provided. Therefore, I would like to approve this study. A few minor points are listed below to help further clarify the authors’ message.

  

- Culturing condition to inactivate Cdc14:
  It was not too clear which temperature (and how long) was used to inactivate Cdc14 in the *cdc14-1* ts mutant. Or, does *cdc14-1* mutant show its meiotic phenotype at an intermediate temperature 30 ^o^C? It would be really helpful if the authors would clarify this point.
- Fig. 3D and 3E:
  In these figures, the Cdc14-GFP signal at the SPB is presented. If I understood correctly, in the pCLB2-3HA-CDC55 setting, SPB duplication does not occur and only one SPB can be found in each cell. If this would be the case, it would be helpful to clearly state so, thus, “asymmetric Cdc14 localisation” is not relevant in this experimental setting.
- Page 8 final paragraph: “…but *symmetrically* during metaphase II” -> asymmetrically
- Fig. 4A:
  Bfa1-tdTomato localization is presented.
  In the “Metaphase II” panel, it would be helpful to have additional small arrowheads in order to indicate SPBs (Spc42-CFP signal-positive dots) without Bfa1-tdTomato signal, so that asymmetric Bfa1-tdTomato localization is easily recognisable.
- Page 12, 2 ^nd^ paragraph:
  It would be helpful to dub the SPB as “Spc42-tdTomato foci” in this paragraph. For example, instead of saying “two SPB” or “four SPB”, say “two Spc42-tdTomato foci” or “Four Spc42-tdTomato foci”. This is because until we see Fig. 7 and 8, we do not know whether a “Spc42-tdTomato focus” represents a non-duplicated SPB (I would call this one SPB) or non-separated SPBs (I would call this two SPBs).
- Figure 7, figure legends:
  Legends corresponding to panels (A-E) contain some inconsistencies, i.e., legends to panel (B) and (E) are missing, and C, D, and E are wrongly assigned.
  In addition, would it be possible to indicate the size/band that corresponds to the Cdc14 protein in the gel in panel F?

 

I have read this submission. I believe that I have an appropriate level of expertise to confirm that it is of an acceptable scientific standard.

## References

[1] IwabuchiMOhsumiKYamamotoTMSawadaWKishimotoT: Residual Cdc2 activity remaining at meiosis I exit is essential for meiotic M-M transition in Xenopus oocyte extracts. *EMBO J*.2000;19(17) : 10.1093/emboj/19.17.4513 4513-23 10.1093/emboj/19.17.4513 10970845PMC302070

[2] IzawaDGotoMYamashitaAYamanoHYamamotoM: Fission yeast Mes1p ensures the onset of meiosis II by blocking degradation of cyclin Cdc13p. *Nature*.2005;434(7032) : 10.1038/nature03406 529-33 10.1038/nature03406 15791259

[3] FoxCZouJRappsilberJMarstonA: Cdc14 phosphatase directs centrosome re-duplication at the meiosis I to meiosis II transition in budding yeast. *Wellcome Open Research*.2017;2: 10.12688/wellcomeopenres.10507.1 10.12688/wellcomeopenres.10507.1 28133632PMC5266553

# Response

**Published:** 2017-02-11

**Authors:** Adele Marston

**Affiliations:** University of Edinburgh, UK

Thank you for your approval of our article, you kind comments and your constructive suggestions. Here we respond to the points made:

- Culturing condition to inactivate Cdc14:
  It was not too clear which temperature (and how long) was used to inactivate Cdc14 in the *cdc14-1* ts mutant. Or, does *cdc14-1* mutant show its meiotic phenotype at an intermediate temperature 30 ^o^C? It would be really helpful if the authors would clarify this point.

*We used 30C, which we believe is efficient at largely inactivating Cdc14 (see comments to other reviewers) as vegetative cells fail to grow at this temperature. We have added this information to the methods. *

- Fig. 3D and 3E:
  In these figures, the Cdc14-GFP signal at the SPB is presented. If I understood correctly, in the pCLB2-3HA-CDC55 setting, SPB duplication does not occur and only one SPB can be found in each cell. If this would be the case, it would be helpful to clearly state so, thus, “asymmetric Cdc14 localisation” is not relevant in this experimental setting.

*We added a statement to this effect in this results section*

- Page 8 final paragraph: “…but *symmetrically* during metaphase II” -> asymmetrically

*This has been corrected.*

  

- Fig. 4A:
  Bfa1-tdTomato localization is presented.
  In the “Metaphase II” panel, it would be helpful to have additional small arrowheads in order to indicate SPBs (Spc42-CFP signal-positive dots) without Bfa1-tdTomato signal, so that asymmetric Bfa1-tdTomato localization is easily recognisable.
- Page 12, 2 ^nd^ paragraph:
  It would be helpful to dub the SPB as “Spc42-tdTomato foci” in this paragraph. For example, instead of saying “two SPB” or “four SPB”, say “two Spc42-tdTomato foci” or “Four Spc42-tdTomato foci”. This is because until we see Fig. 7 and 8, we do not know whether a “Spc42-tdTomato focus” represents a non-duplicated SPB (I would call this one SPB) or non-separated SPBs (I would call this two SPBs).

*This is a very good point. We have changed the text and figure accordingly.*

- Figure 7, figure legends:
  Legends corresponding to panels (A-E) contain some inconsistencies, i.e., legends to panel (B) and (E) are missing, and C, D, and E are wrongly assigned.

*This has been corrected.*

 In addition, would it be possible to indicate the size/band that corresponds to the Cdc14 protein in the gel in panel F?

*There is no obvious band corresponding to Cdc14, a 61 kd protein and as we are unable to confirm that any particular band is Cdc14 we prefer not to annotate this on the gel.*

# Peer review: Referee response for version 1

**DOI:** 10.21956/wellcomeopenres.11325.r19024

**Published:** 2017-01-19

**Authors:** Simonetta Piatti

**Affiliations:** 1 Centre de Recherche en Biologie Cellulaire de Montpellier (CRBM), Montpellier, France

This is an interesting study addressing an important question, i.e. how yeast cells license SPB duplication at the MI/MII transition to be able to form bipolar spindles and support chromosome segregation during reductional division.

 Previous work had shown that the Cdc14 phosphatase must be properly controlled to permit assembly of two MII spindles through an unknown mechanism. In addition, the SPB half-bridge protein Sfi1 has been recently identified as a critical target of Cdc14 in mitosis in order to license SPB duplication.

 In this manuscript the authors show that Cdc14 associates with SPBs also in meiosis. Cdc14 localization at SPBs is asymmetric and requires the Bub2/Bfa1 complex. Cdc14, while not being strictly required for exit from MI (i.e. spindle disassembly), is required for SPB duplication and proper nuclear division in MII. Conversely, premature Cdc14 activation in cells lacking Cdc55 prevents SPB separation in MI. Finally, although SPBs do not duplicate upon Cdc14 inactivation, composition of the SPB remains mostly unchanged.

 Overall, experiments are carefully executed and conclusions are mostly supported by the experimental data. I have, however, a couple of main issues and a few minor points that the authors might want to address.

 Main points:

  

1. The incubation of *cdc14-1* mutant cells at 30°C, which might be a semi-permissive temperature, raises the question as to what extent Cdc14 is actually inactivated in these cells. Wouldn’t it have been better to use a tighter system to inactivate Cdc14 (e.g. expressing *CDC14* from the *CLB2* promoter) ? It is even possible that the Cdc14-1 protein is prematurely released, thereby tempering the effects of temperature inactivation. Has nucleolar release of the Cdc14-1 protein ever been checked at 25°C/30°C ?
  This would also be relevant to interpret correctly the data in Fig. 1B and C, where *cdc14-1* cells do not behave exactly like FEAR mutants.
2. Quantification of SPB signals during meiotic progression (Fig. 7A-E) is an essential piece of data to support the conclusion that *cdc14-1* cells are defective in SPB duplication upon entry into MII. I wonder if a slow-folding fluorescent protein, such as Spc42-tdTomato, is actually a suitable marker for this kind of analyses.
  A related issue concerns the way these data are presented, which I find a bit confusing. For instance, I do not understand why there are gaps in the plot of wild type cells at 0.75-1h and 1.5-1.75h (Fig. 7C), since fluorescence intensities are measured on movies.
  Direct inspection of SPBs by EM, like the authors did, should greatly help understanding what is going on in the *cdc14-1* mutant. However, knowing how many MII cells display the phenotype shown in Fig. 8A is an essential piece of information that is currently missing. Another interesting information is whether SPB over-duplication was ever found in *pCLB2-CDC55* cells.
  Finally, if it were possible to inactivate Cdc14 before commitment to meiosis, would the authors expect a defect in SPB duplication also in MI? The likely possibility that Cdc14 is a crucial licensing factor in ALL kinds of divisions should perhaps be mentioned in the discussion.
3. Since the role of Cdc14 in licensing SPB duplication in mitosis has been linked to Sfi1 dephosphorylation, it would have been nice to check the phosphorylation state of Sfi1 in wild type and *cdc14-1* cells during meiosis. This kind of information might even be extracted from the mass spec data from Fig. 7F.
  Along the same line, it would have been interesting to check if the published unphosphorylatable *sfi1* mutant can suppress the SPB duplication defect of *cdc14-1* cells. This would have offered strong experimental support to the model presented in Fig. 9.

 

 Minor points

  

1. It is unclear why in the movies of *cdc14-1* cells (Fig. 1) spindles do not reassemble in the second meiotic division from the two unduplicated SPBs (like for example in Fig. 8A).
  The abnormal spindle behaviour reported in Fig. 1C is not commented in the text. Surprisingly, *cdc14-1* have a milder phenotype than FEAR mutants in this respect, although *cdc14-1* has a more pronounced defect in spindle disassembly.
2. It is not clear why Spo21 is in the list of Tap-Cdc14 interacting proteins with 0 peptides identified.
3. Cdc14 is reported to localize at SPBs in most *pCLB2-CDC55* cells where Cdc14 is partially or completely released from nucleolus. Yet, in the example cell #2 of Fig. 3D Cdc14 is not visible on the SPB. A better representative image could be selected.
4. Graphs in Fig. 5 and Fig. 6 look pixeled.
5. In Fig. 7C there is a label within the graph that belongs to the IPs underneath. Also, the graphs (C, D, E) are mislabeled relative to the legend.
6. In Fig. 7F two prominent bands appear in the Spc42-Flag IP from wt cells, while one of the two is much decreased in *cdc14-1* cells. Which one of the two is Spc42?

I have read this submission. I believe that I have an appropriate level of expertise to confirm that it is of an acceptable scientific standard, however I have significant reservations, as outlined above.

# Response

**Published:** 2017-02-11

**Authors:** Adele Marston

**Affiliations:** University of Edinburgh, UK

Thank you for your approval in principle of our article and you helpful suggestions, here we have addressed your reservations:

 Main points:

  

1. The incubation of *cdc14-1* mutant cells at 30°C, which might be a semi-permissive temperature, raises the question as to what extent Cdc14 is actually inactivated in these cells. Wouldn’t it have been better to use a tighter system to inactivate Cdc14 (e.g. expressing *CDC14* from the *CLB2* promoter) ? It is even possible that the Cdc14-1 protein is prematurely released, thereby tempering the effects of temperature inactivation. Has nucleolar release of the Cdc14-1 protein ever been checked at 25°C/30°C ?
  This would also be relevant to interpret correctly the data in Fig. 1B and C, where *cdc14-1* cells do not behave exactly like FEAR mutants.

*Please see response to comment by Soni Lacefield. We agree we cannot completely rule out partial inactivation of Cdc14.*

1. Quantification of SPB signals during meiotic progression (Fig. 7A-E) is an essential piece of data to support the conclusion that *cdc14-1* cells are defective in SPB duplication upon entry into MII. I wonder if a slow-folding fluorescent protein, such as Spc42-tdTomato, is actually a suitable marker for this kind of analyses.

*Because of these and other concerns (see response to Soni Lacefield) we used EM as a completely independent way to examine SPB duplication in cdc14-1 mutants. We believe the EM data provides convincing evidence for our conclusions, while the fluorescence intensity measurements provide some supporting data.*

 A related issue concerns the way these data are presented, which I find a bit confusing. For instance, I do not understand why there are gaps in the plot of wild type cells at 0.75-1h and 1.5-1.75h (Fig. 7C), since fluorescence intensities are measured on movies.

*The data for individual cells was lined up so that the 1h timepoint is defined as the point at which 2 SPBs are first observed. The different coloured lines represent 1, 2 or 4 SPBs. We have clarified this point in the figure legend.*

 Direct inspection of SPBs by EM, like the authors did, should greatly help understanding what is going on in the *cdc14-1* mutant. However, knowing how many MII cells display the phenotype shown in Fig. 8A is an essential piece of information that is currently missing. Another interesting information is whether SPB over-duplication was ever found in *pCLB2-CDC55* cells.

*Overall we observed SPBs in 8 cdc14-1 cells by EM. Out of these, evidence of late meiosis II events (i.e. spore formation/vesicles) was observed in 3 of these. The image shown in Figure 8A is one example of two in which a spindle could be followed between 2SPBs. The other example was spread over >8 sections (spore formation undetermined) so the image shown was favoured. The third example showed the spore wall forming around a nucleus so SPBs were not connected by spindles in that case.*

*No evidence of overduplication of SPBs was observed in pCLB2-CDC55 cells (n=6).*

*We have included this information in the relevant section in the paper. All EM images are available here: *https://osf.io/g5cmh/ ( Marston, 2016).

 Finally, if it were possible to inactivate Cdc14 before commitment to meiosis, would the authors expect a defect in SPB duplication also in MI? The likely possibility that Cdc14 is a crucial licensing factor in ALL kinds of divisions should perhaps be mentioned in the discussion.

*The licensing event that enables SPB duplication during meiosis I would be expected to occur in the last stages of vegetative growth i.e. at mitotic exit prior to commitment to meiosis. So yes, it would be required in the same way that Cdc14 is required to license SPBs during vegetative growth.*

 2.. Since the role of Cdc14 in licensing SPB duplication in mitosis has been linked to Sfi1 dephosphorylation, it would have been nice to check the phosphorylation state of Sfi1 in wild type and *cdc14-1* cells during meiosis. This kind of information might even be extracted from the mass spec data from Fig. 7F.

*Unfortunately we did not detect Sfi1 phosphorylation in our quantitative mass spec analysis. We attempted to examine Sfi1 phosphorylation on western blots and phostag gels but since the time period where Sfi1 dephosphorylation is expected to be observed (anaphase I) is very short, we were unable to obtain sufficient time resolution to make firm conclusions about the effect of Cdc14 inactivation of Sfi1 phosphorylation status.*

 3. Along the same line, it would have been interesting to check if the published unphosphorylatable *sfi1* mutant can suppress the SPB duplication defect of *cdc14-1* cells. This would have offered strong experimental support to the model presented in Fig. 9.

*We agree that this would be a very interesting experiment but despite extensive efforts we were unable to generate yeast strains carrying the Sfi1 phosphonull mutants and were therefore sadly unable to do this experiment. Diploid cells carrying the mutations as the sole copy of Sfi1 were extremely sick and would not enter meiosis. Other approaches were also attempted, for example expression of Sfi1 only in meiosis but due to both biological and technical reasons we have so far been unable to address this important point.*

 Minor points

  

1. It is unclear why in the movies of *cdc14-1* cells (Fig. 1) spindles do not reassemble in the second meiotic division from the two unduplicated SPBs (like for example in Fig. 8A).

*For reasons we do not understand, spindles reassemble in a fraction of cdc14-1 cells, depending on the growth condition. In live cell imaging (Fig. 1), about 10% of cells were observed to reassemble spindles, however, the fraction is likely to be higher in flasks (Fig. 8A).*

 The abnormal spindle behaviour reported in Fig. 1C is not commented in the text. Surprisingly, *cdc14-1* have a milder phenotype than FEAR mutants in this respect, although *cdc14-1* has a more pronounced defect in spindle disassembly.

*We have included a sentence raising this point in the results section.*

 2. It is not clear why Spo21 is in the list of Tap-Cdc14 interacting proteins with 0 peptides identified.

*For completeness, all SPB proteins were included in the list, regardless of whether they were identified in the mass spectrometer.*

 3. Cdc14 is reported to localize at SPBs in most *pCLB2-CDC55* cells where Cdc14 is partially or completely released from nucleolus. Yet, in the example cell #2 of Fig. 3D Cdc14 is not visible on the SPB. A better representative image could be selected.

*Cell #3 shows a cell where Cdc14 is on the SPB. Cell #2 is an example of where the diffuse released Cdc14 signal overlaps with the SPB. As these are the criteria that were scored, these examples are appropriate.*

 4. Graphs in Fig. 5 and Fig. 6 look pixeled.

*We have uploaded new versions of these figures that should correct this problem.*

 5. In Fig. 7C there is a label within the graph that belongs to the IPs underneath. Also, the graphs (C, D, E) are mislabeled relative to the legend.

*We have corrected this.*

 6. In Fig. 7F two prominent bands appear in the Spc42-Flag IP from wt cells, while one of the two is much decreased in *cdc14-1* cells. Which one of the two is Spc42?

*The top band, which has similar intensity in the two samples, is Spc42-Flag*

# Peer review: Referee response for version 1

**DOI:** 10.21956/wellcomeopenres.11325.r19025

**Published:** 2017-01-16

**Authors:** Fernando Monje-Casas

**Affiliations:** 1 CABIMER (Andalusian Molecular Biology and Regenerative Medicine Centre), The Spanish National Research Council (CSIC), Seville, Spain

In their manuscript, Fox *et al.* demonstrate that Cdc14, a key phosphatase that promotes CDK inactivation and exit from mitosis in *Saccharomyces cerevisiae*, also plays a pivotal role in promoting the re-licensing of the SPBs at the meiosis I to meiosis II transition, thus ensuring the re-duplication of these structures between these two different meiotic phases. In order to fulfill this function, Cdc14 is loaded on a single SPB during anaphase I in a process that depends on the Bfa1/Bub2 complex. Once loaded on the SPB, Cdc14 likely promotes dephosphorylation of key substrates on this structure, thereby allowing the re-duplication of the SPBs in meiosis II. This newly suggested meiotic function of Cdc14 helps to shed light on how cells establish the particular pattern of chromosome segregation during meiosis, an essential cell division process by which gametes are generated. Overall, the experiments detailed in the manuscript are carefully designed, nicely presented and well executed. Also, and importantly, the final conclusions of the manuscript are properly sustained by the provided experimental data. Therefore, I support an approved status for this article. In any case, I also indicate some minor corrections that would need to be introduced, as well as some suggestions that might help the authors to improve the manuscript and strengthen some of the conclusions:

  

1. In page 8, when the authors indicate that *slk19*∆ and *spo12*∆ cells disassembled anaphase I spindles with a timing comparable to wild type cells, they only refer to Figure 1B, but Figure 1A should be also mentioned. Additionally, and regarding the data in Figure 1A, I would suggest to show images for other live cell experiments that are more representative of the previous statement. Although Figure 1B indeed shows that, as an average, time from anaphase I onset until spindle disassembly is similar for wild type, *slk19*∆ and *spo12*∆ cells, in the images shown in Figure 1A for the *slk19*∆ and *spo12*∆ cells the spindle disassembles, respectively, 20 min and 30 min after anaphase I onset, which is significantly earlier than for the wild type (40 min).
2. Also in page 8, it is later stated that “[…] the two component GAP Bfa1/Bfa1, […] localizes symmetrically at SPBs during metaphase I, anaphase I but symmetrically during metaphase II”. This sentence should be corrected, since, as shown in Figure 1B, Bfa1 localization is asymmetric during metaphase II.
3. The localization of Cdc14-GFP is difficult to assess in Figure 3A, due to the nucleolar background and the appearance of other GFP foci that do not co-localize with the SPBs and whose nature it is not indicated ( *e.g.*, 1:15 time point in Figure 3A). Since the authors show different proteins that interact with Cdc14 on the SPBs, they might consider to use a different approach if they plan to further analyze this localization in the future. As such, a bimolecular fluorescence complementation assay (BiFC) ^1^ could be helpful to determine the exact timing and pattern of localization of Cdc14 on the SPBs. The BiFC assay could facilitate tracking Cdc14 localization exclusively to the SPBs by means of the reconstitution of a fluorescent signal when the phosphatase and a SPB component, both tagged with different fragments of a fluorescent protein, interact.
4. In page 16, when the authors show a 1.5-fold increase in Spc42-tdTomato intensity as wild type cells progressed from G1 into prophase I, they only refer to Figure 7A, but it is in Figure 7B where the quantification is shown.
5. A shocking observation is that, despite Bfa1/Bub2 being symmetrically localized in anaphase I and Cdc14 activity being presumably required at both SPBs to trigger their duplication in meiosis II, the phosphatase is asymmetrically localized to only one SPB during anaphase I. This is an obvious caveat and, although I do appreciate that it would require further extensive analysis for this question to be solved, the authors might have speculated a little more extensively about the reasons for this asymmetry in the discussion of the manuscript.
6. A prediction of the model proposed by Fox *et al*. is that re-duplication of the SPBs should be restored in a *cdc14-1* mutant background by conditionally forcing Cdc14 loading on the SPBs at the appropriate time ( *e.g.*, by temporarily expressing a fusion between Cdc14 and a SPB component). This is a relatively straightforward experiment that the authors might consider to carry out at some point to further strengthen their model. Furthermore, and coming back to the differential localization of Cdc14 on the SPBs during anaphase I, this experiment could be informative to address the importance of this asymmetry in future studies. Cdc14 could be fused either to Bfa1 (asymmetrically localized) or an integral SPB component (symmetrically localized), and then analyze the consequences of expressing both Cdc14 fusions during meiosis.

I have read this submission. I believe that I have an appropriate level of expertise to confirm that it is of an acceptable scientific standard.

## References

[1] SungMKHuhWK: Bimolecular fluorescence complementation analysis system for in vivo detection of protein-protein interaction in Saccharomyces cerevisiae. *Yeast*.2007;24(9) : 10.1002/yea.1504 767-75 10.1002/yea.1504 17534848

# Response

**Published:** 2017-02-11

**Authors:** Adele Marston

**Affiliations:** University of Edinburgh, UK

*We are grateful for your approval of our manuscript, kind comments and helpful suggestions, which we respond to here.*

 1. In page 8, when the authors indicate that *slk19*∆ and *spo12*∆ cells disassembled anaphase I spindles with a timing comparable to wild type cells, they only refer to Figure 1B, but Figure 1A should be also mentioned. Additionally, and regarding the data in Figure 1A, I would suggest to show images for other live cell experiments that are more representative of the previous statement. Although Figure 1B indeed shows that, as an average, time from anaphase I onset until spindle disassembly is similar for wild type, *slk19*∆ and *spo12*∆ cells, in the images shown in Figure 1A for the *slk19*∆ and *spo12*∆ cells the spindle disassembles, respectively, 20 min and 30 min after anaphase I onset, which is significantly earlier than for the wild type (40 min).

*We have now included the reference to Figure 1A. Under the conditions we observed them, the timing of spindle disassembly varied across all strains *(Figure 1B) *, we tried to show representative images to reflect this.*

**

*2. *Also in page 8, it is later stated that “[…] the two component GAP Bfa1/Bfa1, […] localizes symmetrically at SPBs during metaphase I, anaphase I but symmetrically during metaphase II”. This sentence should be corrected, since, as shown in Figure 1B, Bfa1 localization is asymmetric during metaphase II.

*The typo has been corrected.*

 3. The localization of Cdc14-GFP is difficult to assess in Figure 3A, due to the nucleolar background and the appearance of other GFP foci that do not co-localize with the SPBs and whose nature it is not indicated ( *e.g.*, 1:15 time point in Figure 3A). Since the authors show different proteins that interact with Cdc14 on the SPBs, they might consider to use a different approach if they plan to further analyze this localization in the future. As such, a bimolecular fluorescence complementation assay (BiFC) ^1^ could be helpful to determine the exact timing and pattern of localization of Cdc14 on the SPBs. The BiFC assay could facilitate tracking Cdc14 localization exclusively to the SPBs by means of the reconstitution of a fluorescent signal when the phosphatase and a SPB component, both tagged with different fragments of a fluorescent protein, interact.

*It is presently unclear what the non-nucleolar, non-SPB Cdc14-GFP foci represent. Although outside the scope of the current manuscript, we appreciate the excellent suggestion to use a bimolecular complementation assay to probe the co-localization of Cdc14 with SPBs. It would be particularly interesting to determine whether Cdc14 can be detected at both SPBs using this system.*

**

*4. *In page 16, when the authors show a 1.5-fold increase in Spc42-tdTomato intensity as wild type cells progressed from G1 into prophase I, they only refer to Figure 7A, but it is in Figure 7B where the quantification is shown.

*This has been corrected.*

 5. A shocking observation is that, despite Bfa1/Bub2 being symmetrically localized in anaphase I and Cdc14 activity being presumably required at both SPBs to trigger their duplication in meiosis II, the phosphatase is asymmetrically localized to only one SPB during anaphase I. This is an obvious caveat and, although I do appreciate that it would require further extensive analysis for this question to be solved, the authors might have speculated a little more extensively about the reasons for this asymmetry in the discussion of the manuscript.

*We agree, this is indeed a surprising finding that remains unexplained. We expanded our discussion as suggested, though ultimately this is indeed a very puzzling observation for which we currently do not have an explanation.*

 6. A prediction of the model proposed by Fox *et al*. is that re-duplication of the SPBs should be restored in a *cdc14-1* mutant background by conditionally forcing Cdc14 loading on the SPBs at the appropriate time ( *e.g.*, by temporarily expressing a fusion between Cdc14 and a SPB component). This is a relatively straightforward experiment that the authors might consider to carry out at some point to further strengthen their model. Furthermore, and coming back to the differential localization of Cdc14 on the SPBs during anaphase I, this experiment could be informative to address the importance of this asymmetry in future studies. Cdc14 could be fused either to Bfa1 (asymmetrically localized) or an integral SPB component (symmetrically localized), and then analyze the consequences of expressing both Cdc14 fusions during meiosis.

*We attempted this experiment using the GFP-GBP system. However, we were unable to obtain strains in which Cdc14 and SPB components were both tagged with these binding partners. Although other explanations are possible, these findings are consistent with the idea that forced constitutive targeting of Cdc14 to the SPB is lethal also in vegetative cells. Due to the lethality we were unfortunately unable to assess the effect on meios*is.

# Peer review: Referee response for version 1

**DOI:** 10.21956/wellcomeopenres.11325.r19026

**Published:** 2017-01-16

**Authors:** Soni Lacefield

**Affiliations:** 1 Department of Biology, Indiana University Bloomington, Bloomington, IN, USA

This manuscript addresses the intriguing and poorly understood question of how cells regulate the transition from meiosis I to meiosis II. During this transition, cells must disassemble the meiosis I spindle, duplicate their spindle poles, and undergo a second round of spindle assembly without re-replicating their DNA.  Previous work established that Cdc14 is  important for this transition because cells lacking Cdc14 only formed one spindle, but it's role had not been characterized. This work uses a combination of fluorescence microscopy, electron microscopy, and proteomics to demonstrate  that Cdc14 SPB localization is required for the re-licensing of SPBs in the meiosis I to meiosis II transition. Furthermore, the proper timing of Cdc14 is also important, as premature Cdc14 activity results in a failure of SPBs to separate. 

 Overall, this is an important study that identifies a novel role for Cdc14 and increases our understanding of how SPBs duplicate between the two divisions. However, there are some points that need addressed:

 Major considerations:

1. Does the Cdc14-1 allele result in a complete loss of function in meiosis? A discussion of whether this is a hypomorph or complete loss of function would be helpful, especially for thinking about the conclusion that "Cdc14 is not absolutely required for CDK down-regulation at meiosis I exit".
2. In Figure 1 C, why do only 50% of the cells form 2 spindles instead of all of the cells? The duration of the movies needs to be increased to ensure that there is enough time for meiosis II to occur.
3. I am having difficulty reconciling the results in Fig 7 with the model in Fig. 9. Why does the Spc42 fluorescence remain the same throughout meiosis I (metaphase I and anaphase I) when the SPB should be duplicating during the time? It would be helpful to have a graph showing the change in Spc42 fluorescence, tracking individual cells, also marking the time of anaphase I and anaphase II.
4. Does Sfi1 phosphorylation change throughout meiosis? Does Sfi1 remain phosphorylated in the Cdc14-1 cells?
5. A thought about the conclusions: The prematurely released Cdc14 in pCLB2-CDC55 cells also localized to SPBs but does not allow SPBs reduplication. Does this finding suggest that the Cdc14 SPB localization is required but not sufficient to promote SPB duplication if the SPBs have not first separated?

    

 Minor points:

1. The last sentence in the abstract should end in "meiosis II transition." It currently says meiosis I.
2. The sentence in the last paragraph on page 8 should read, "An exception is the two component GAP Bub2/Bfa1 which localized symmetrically at SPBs during metaphase I, anaphase I by asymmetrically during metaphase II".
3. The reference to the white arrow in Figure 8 is right after talking about vesicle formation, but the arrow does not point at the vesicles.
4. On page 16, the reference to Fig. 7F is missing and one of the references to 7E should be to 7G. 

I have read this submission. I believe that I have an appropriate level of expertise to confirm that it is of an acceptable scientific standard, however I have significant reservations, as outlined above.

# Response

**Published:** 2017-02-11

**Authors:** Adele Marston

**Affiliations:** University of Edinburgh, UK

*We are grateful for your approval in principle and constructive comments. Here we address your reservations.*

 Major considerations:

 1. Does the Cdc14-1 allele result in a complete loss of function in meiosis? A discussion of whether this is a hypomorph or complete loss of function would be helpful, especially for thinking about the conclusion that "Cdc14 is not absolutely required for CDK down-regulation at meiosis I exit".

*It is true that we cannot be sure that the cdc14-1 allele is completely inactivated during meiosis. Our attempts to deplete or degrade Cdc14 during meiosis have been unsuccessful and therefore temperature-sensitive alleles are the best tools we have. We find that spindle disassembly is impaired in cdc14-1 conditions in mitosis but not meiosis under similar temperature conditions, leading us to suggest a lesser requirement for Cdc14 in meiotic spindle disassembly. Recently, similar findings have been reported for meiosis II using a different temperature sensitive allele *(Argüello-Miranda *et al., *2017). *We have revised the text to include the possibility that Cdc14 retains partial function using these alleles and included a reference to this recent study.*

 2. In Figure 1 C, why do only 50% of the cells form 2 spindles instead of all of the cells? The duration of the movies needs to be increased to ensure that there is enough time for meiosis II to occur.

*The duration of the movies was 12h, however not all cells complete meiosis under the imaging conditions used for this experiment. Rather than bias the data by scoring only cells that complete meiosis II, all cells that enter meiosis were included in the analysis. This experiment was performed at a time where we were still establishing optimal conditions for live cell imaging of cells undergoing meiosis. Although we recognise that we could improve on this with our optimised conditions (where essentially all cells complete meiosis), we feel that the data presented clearly shows the effect of different mutants on meiosis I spindle disassembly.*

 3. I am having difficulty reconciling the results in Fig 7 with the model in Fig. 9. Why does the Spc42 fluorescence remain the same throughout meiosis I (metaphase I and anaphase I) when the SPB should be duplicating during the time? It would be helpful to have a graph showing the change in Spc42 fluorescence, tracking individual cells, also marking the time of anaphase I and anaphase II.

*SPB fluorescence stays the same throughout meiosis I (metaphase I and anaphase I) because SPBs are already duplicated before metaphase I (i.e. in the ndt80* *D* *arrest – see Figure 7C). We do note, however, that a greater increase in fluorescence might be expected during the later stages of anaphase I in wild type cells. The source data for this experiment that would allow the reader to make traces of individual cells is available at *https://osf.io/g5cmh/ ( Marston, 2016). *The fluorescence analysis of Spc42-tdTomato is complicated by two factors that mean we cannot make stand alone conclusions from it. First, in other experiments (not shown) we concluded that Spc42 incorporation into the SPB is continuous and exchange occurs. Second, the SPB undergoes a remodelling and maturation during meiosis II. As a result of these concerns we performed electron microscopy, which clearly show a failure in SPB re-duplication in *cdc14-1 *mutants.* *We believe that the EM data unequivocally supports our conclusions, while the fluorescence intensity data offer supportive, but not definitive evidence.*

 2. Does Sfi1 phosphorylation change throughout meiosis? Does Sfi1 remain phosphorylated in the Cdc14-1 cells?

*Despite extensive efforts to examine Sfi1 phosphorylation in wild type and cdc14-1 mutants undergoing meiosis, the findings were inconclusive. Judging by the presence of multiple Sfi1 species on western blots, Sfi1 appears to be extensively phosphorylated during meiosis in wild type cells. Our model predicts transient dephosphorylation at the time of anaphase I, while phosphorylation should persist in cdc14-1 mutants. Unfortunately, using this method, we were unable to reproducibly visualize Sfi1 dephosphorylation during anaphase I during wild type cells, likely due to the very short time window in which it occurs and variable cell synchronization, even using the best methods currently available (NDT80 block-release).*

**

*3. *A thought about the conclusions: The prematurely released Cdc14 in *pCLB2-CDC55 cells* also localized to SPBs but does not allow SPBs reduplication. Does this finding suggest that the Cdc14 SPB localization is required but not sufficient to promote SPB duplication if the SPBs have not first separated?

*SPBs undergo the first round of duplication in pCLB2-CDC55 cells but do not appear to be able to separate. The licensing model predicts that, following Cdc14 dephosphorylation of Sfi1, CDK activity is required to separate the SPBs. We suggest that Cdc14 at the SPB counteracts CDK activity, thereby preventing separation of SPBs. Therefore, we agree, SPB separation may be a pre-requisite for duplication.*

 Minor points:

1. The last sentence in the abstract should end in "meiosis II transition." It currently says meiosis I.
*We have corrected this* 
2. The sentence in the last paragraph on page 8 should read, "An exception is the two component GAP Bub2/Bfa1 which localized symmetrically at SPBs during metaphase I, anaphase I by asymmetrically during metaphase II".
*Corrected.*
3. The reference to the white arrow in Figure 8 is right after talking about vesicle formation, but the arrow does not point at the vesicles.
   
*The arrows mark the SPBs, not the vesicles, as written in the figure legend. We made this clearer by writing “SPB1, white arrow”, “SPB2, white arrow”.*
4. On page 16, the reference to Fig. 7F is missing and one of the references to 7E should be to 7G. 

*We have corrected this.*
